# Continuous remission of single-mode therapy with pembrolizumab plus chemotherapy in locally advanced hypopharyngeal carcinoma: a case report

**DOI:** 10.3389/fimmu.2025.1557565

**Published:** 2025-06-05

**Authors:** Guoning Yu, Jianqiao He, Yingna Gao, Xiaoqiong Shi, Yi Ma, Shicai Chen, Hongliang Zheng, Minhui Zhu, Caiyun Zhang

**Affiliations:** ^1^ Department of Otorhinolaryngology-Head and Neck Surgery, Changhai Hospital, Naval Medical University, Shanghai, China; ^2^ The 909th Hospital, School of Medicine, Xiamen University, Zhangzhou, China

**Keywords:** hypopharyngeal cancer, monoimmunotherapy, pembrolizumab, PD-1, sustained survival benefit, case report

## Abstract

Hypopharyngeal carcinoma, one of the common malignant tumors of the head and neck, is associated with high tumor aggressiveness, early cervical lymph node metastasis, and a poor prognosis. Neoadjuvant immunotherapy has been gradually introduced to treat locally advanced head and neck squamous cell carcinoma (LA-HNSCC), including hypopharyngeal carcinoma. Despite survival benefit advantages, there is no consensus on the treatment mode after neoadjuvant immunotherapy, especially for patients achieving a complete response (CR). It remains uncertain whether surgery, radical radiotherapy, or maintenance with immunotherapy should be chosen for patients achieving CR. Moreover, there are no reports of the successful use of monoimmunotherapy as maintenance therapy in patients who achieve CR with neoadjuvant immunotherapy. Here, we present the case of an older woman diagnosed with locally advanced hypopharyngeal carcinoma with cervical esophageal involvement who presented with dyspnea and swallowing obstruction. After 18 courses of weekly paclitaxel + carboplatin combined with cetuximab (PCC), during which she received pembrolizumab every 3 weeks, the patient’s laryngoscopy and radiologic imaging results revealed that she had achieved CR. She was subsequently maintained with pembrolizumab alone, and no tumor recurrence was observed on multiple examinations during follow-up. No surgery or radiotherapy was performed. From the beginning of treatment to the present (21 months), the patient’s general condition and quality of life improved significantly (Karnofsky performance status [KPS] = 100), and laryngeal function was well preserved. Our results indicate that patients who achieve CR after neoadjuvant immunotherapy may be maintained with immunotherapy (with surgery or radiotherapy as a salvage measure), which can improve disease-free survival in patients with relatively normal laryngeal function. This single-mode treatment may achieve long-term survival in some LA-HNSCC patients.

## Introduction

The prognosis of LA-HNSCC patients is poor, and hypopharyngeal carcinoma has one of the worst prognoses, characterized by a high degree of aggressiveness, hidden location of onset, atypical early symptoms, and susceptibility to cervical lymph node metastasis. The five-year overall survival (OS) rate is only 30–35% (1). The treatment of locally advanced hypopharyngeal carcinoma is limited: radical surgery often severely damages or even sacrifices laryngeal function, as does radical radiotherapy At present, for locally advanced hypopharyngeal cancer, the National Comprehensive Cancer Network (NCCN) guidelines also recommend induction chemotherapy as an important treatment choice (2). Induction chemotherapy can reduce the tumor stage and surgery scope, increase the rate of larynx-preserving surgery, and provide other advantages; however, the improvement in patient survival is limited in large-scale data analyses.

Immunotherapy has become the first-line treatment for recurrent/metastatic head and neck squamous cell carcinoma (R/M-HNSCC). Immunotherapy can achieve antitumor effects by regulating the immune system, greatly affecting the treatment prospects of cancer patients (3, 4). Squamous cell carcinoma of the head and neck is a solid tumor with a complex tumor microenvironment (TME), and abundant immune cell infiltration into the TME enables the efficacy of immunotherapy (5, 6). The Food and Drug Administration (FDA) approved nivolumab and pembrolizumab for treating R/M-HNSCC patients in 2019, and their efficacy was also verified in real-world clinical settings. However, the application of immunotherapy for treating LA-HNSCC is still in the exploratory stage.

Preoperative neoadjuvant immunotherapy in patients with LA-HNSCC is also gradually being applied in relevant clinical research. To date, many studies have shown that patients who achieve major pathological response (MPR) or even complete pathological response (pCR) after neoadjuvant immunotherapy have a better prognosis, suggesting that neoadjuvant immunotherapy can not only control the disease and preserve organ function in some patients but also prolong patient survival (7). However, the specific treatment model is not yet unified; for example, for LA-HNSCC patients who receive neoadjuvant immunotherapy and achieve CR, should we proceed with radical surgery or radical radiotherapy? Or should we continue to maintain immunotherapy without surgery or radiation therapy (surgery or radiation therapy as salvage treatment)? To date, the guidelines on this issue are not clear. The current evidence of immunotherapy in patients with HNSCC has mainly been obtained from R/M-HNSCC studies, and clinical studies on immunotherapy for patients with LA-HNSCC are ongoing. There are no reports on single-mode therapy with neoadjuvant immunotherapy for patients with LA-HNSCC, and potential molecular markers that can predict the benefits of immunotherapy remain lacking.

**Figure 1:**
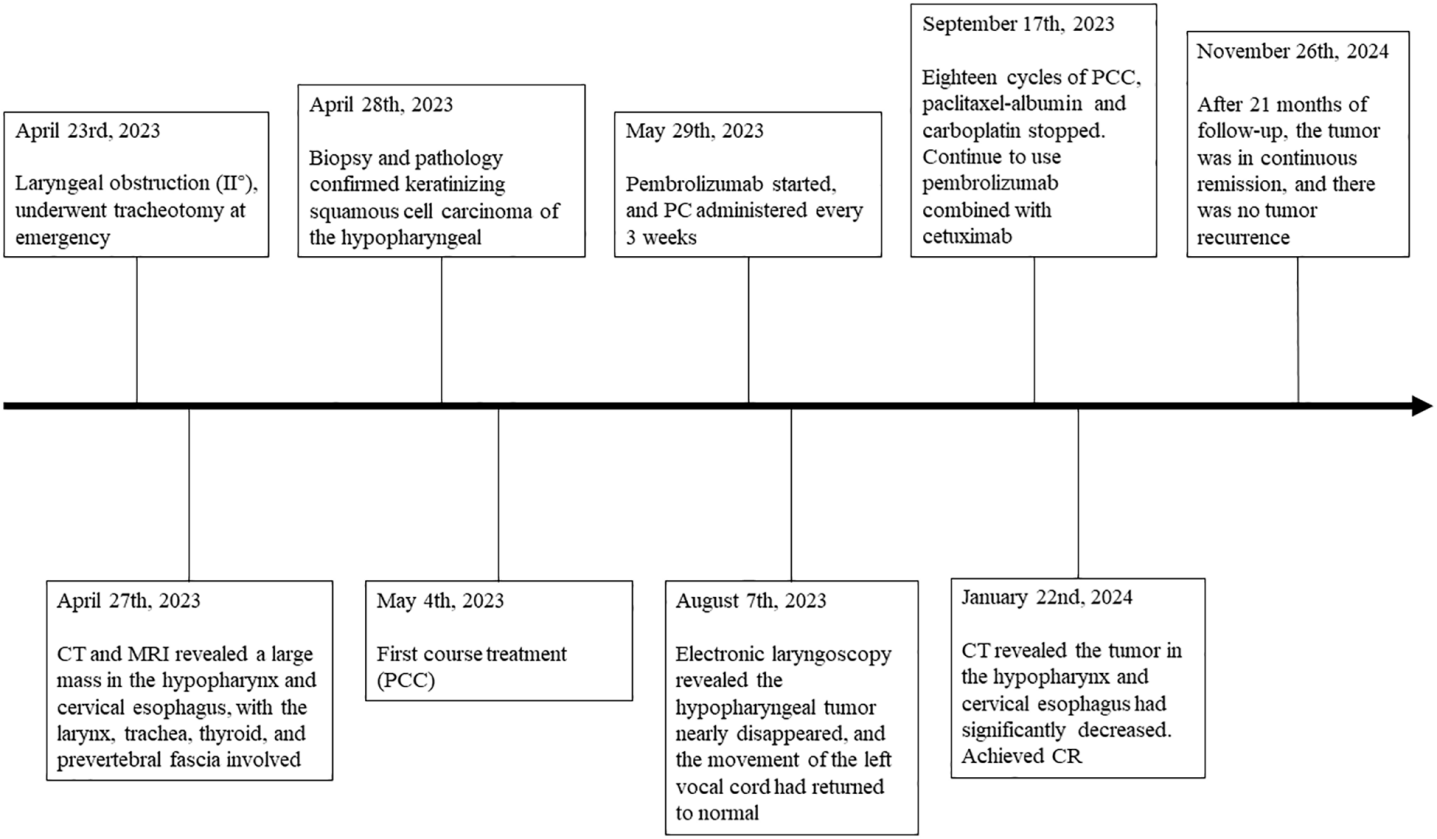
Timeline of the patient’s clinical course. Q3w, 3 weeks using a dose; C, cycle; CR, complete response; PCC, paclitaxel-albumin + carboplatin + cetuximab; PC, pembrolizumab + cetuximab.

Here, we report the case of a patient with locally advanced hypopharyngeal carcinoma involving the cervical esophagus. After a total of 18 courses of weekly paclitaxel plus carboplatin chemotherapy combined with cetuximab (PCC) were given, during which the patient received pembrolizumab every 3 weeks, a complete response (CR) was achieved at the end of neoadjuvant immunochemotherapy. Subsequent maintenance therapy with pembrolizumab plus cetuximab was given every 3 weeks (**Figure 1**). To date, there has been no recurrence within 21 months. The tracheal cannula was removed, and the voice function and respiratory condition returned to normal. The patient’s mental state significantly improved, and their body weight increased. During treatment, no severe adverse events occurred except for a mild rash. The successful use of single-mode neoadjuvant immunochemotherapy in locally advanced hypopharyngeal carcinoma might provide new ideas and directions for treating LA-HNSCC.

## Case presentation

**Figure 2:**
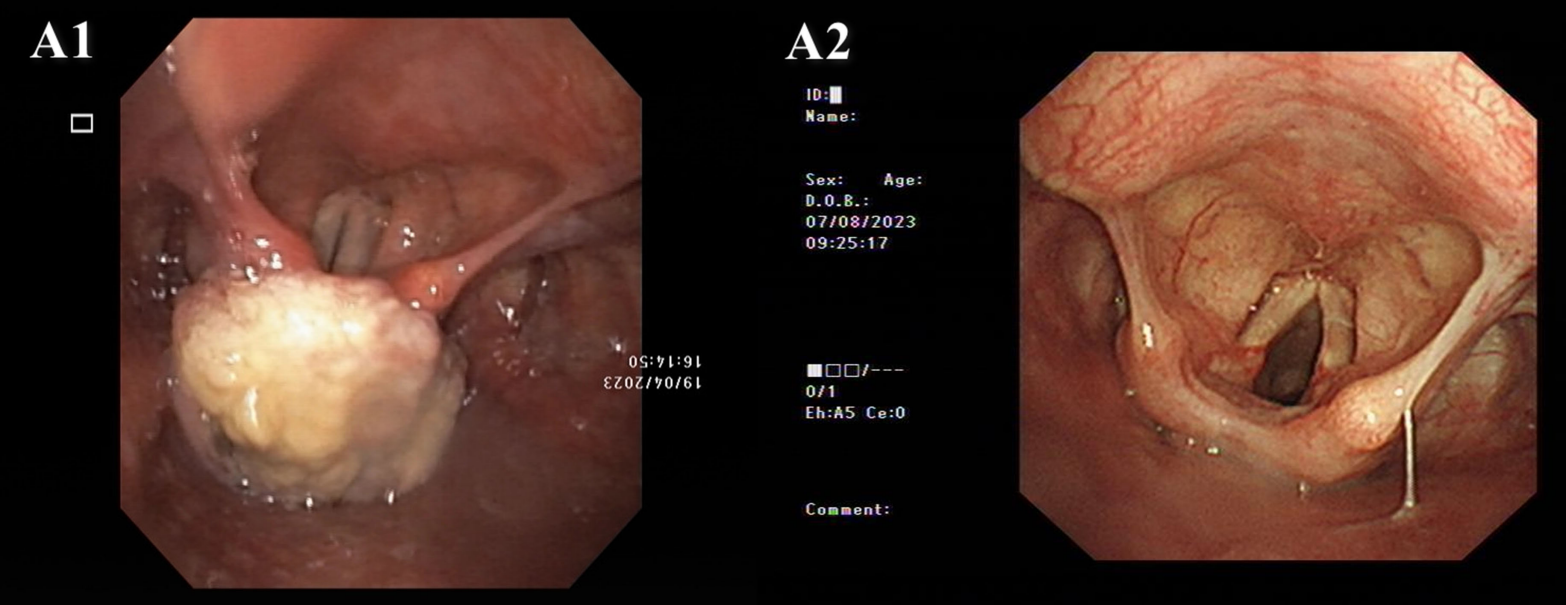
Electronic laryngoscope images. A1. Pretreatment: A large mass in the postcricoid area of the hypopharynx. Both vocal cords were smooth, but fixed. A2. Posttreatment (August 8th, 2023): The hypopharyngeal tumor had nearly disappeared, and movement of the left vocal cord returned to normal.

A 69-year-old woman with “swallowing obstruction for more than 2 months and dyspnea for more than 10 days” was admitted to the emergency department of otorhinolaryngology and head and neck surgery at our hospital on April 23, 2023. Before the visit, an electronic laryngoscopic examination revealed a large mass in the postcricoid area of the hypopharynx, and the bilateral vocal cords were smooth and fixed (**Figure 2**).

**Figure 3:**
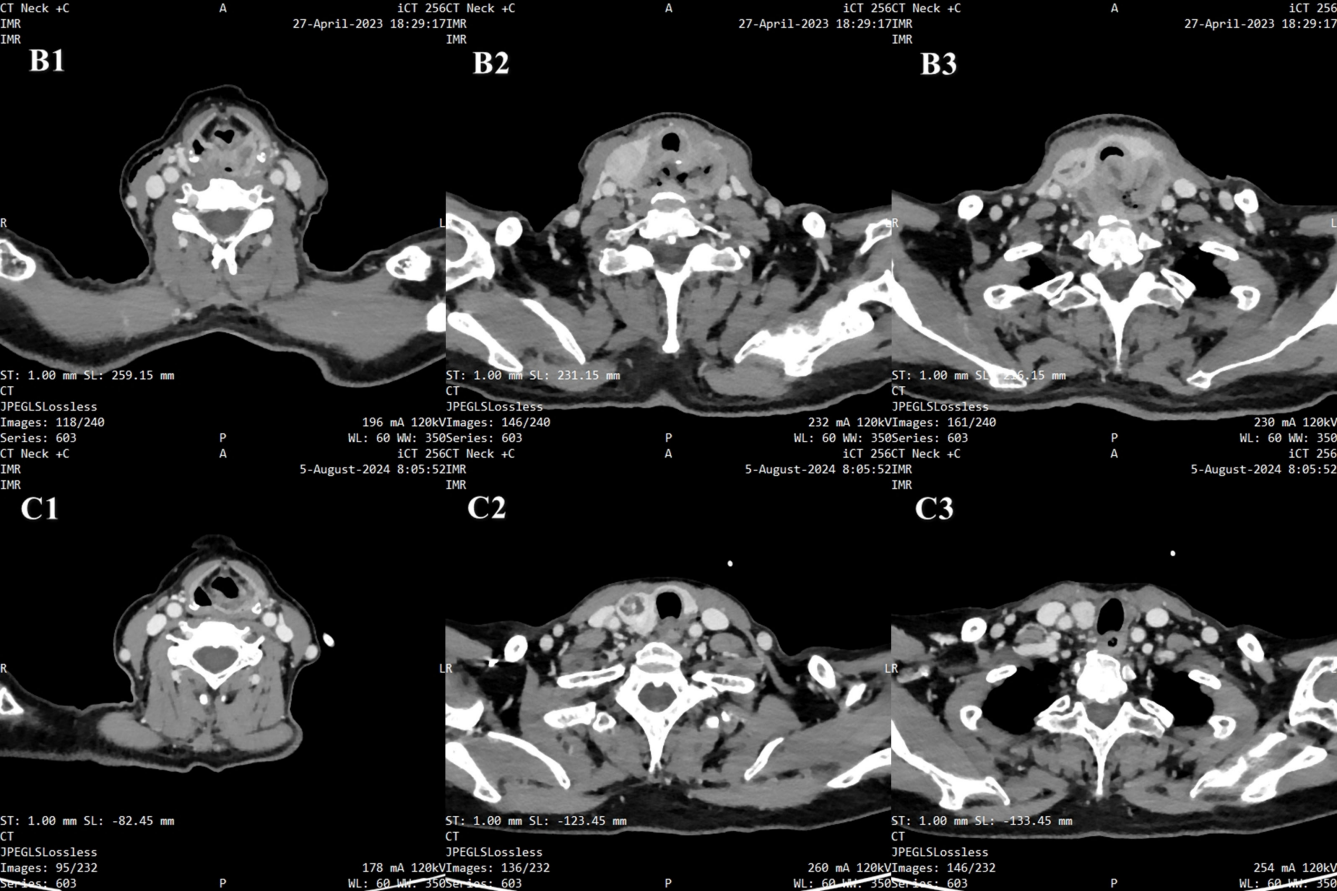
Radiographic images. B1-B3. Pretreatment: A large mass in the hypopharynx and cervical esophagus, with the larynx, trachea, thyroid, and prevertebral fascia involved. C1-C3. Posttreatment: The number of tumors in the hypopharynx and cervical esophagus significantly decreased.

**Figure 4:**
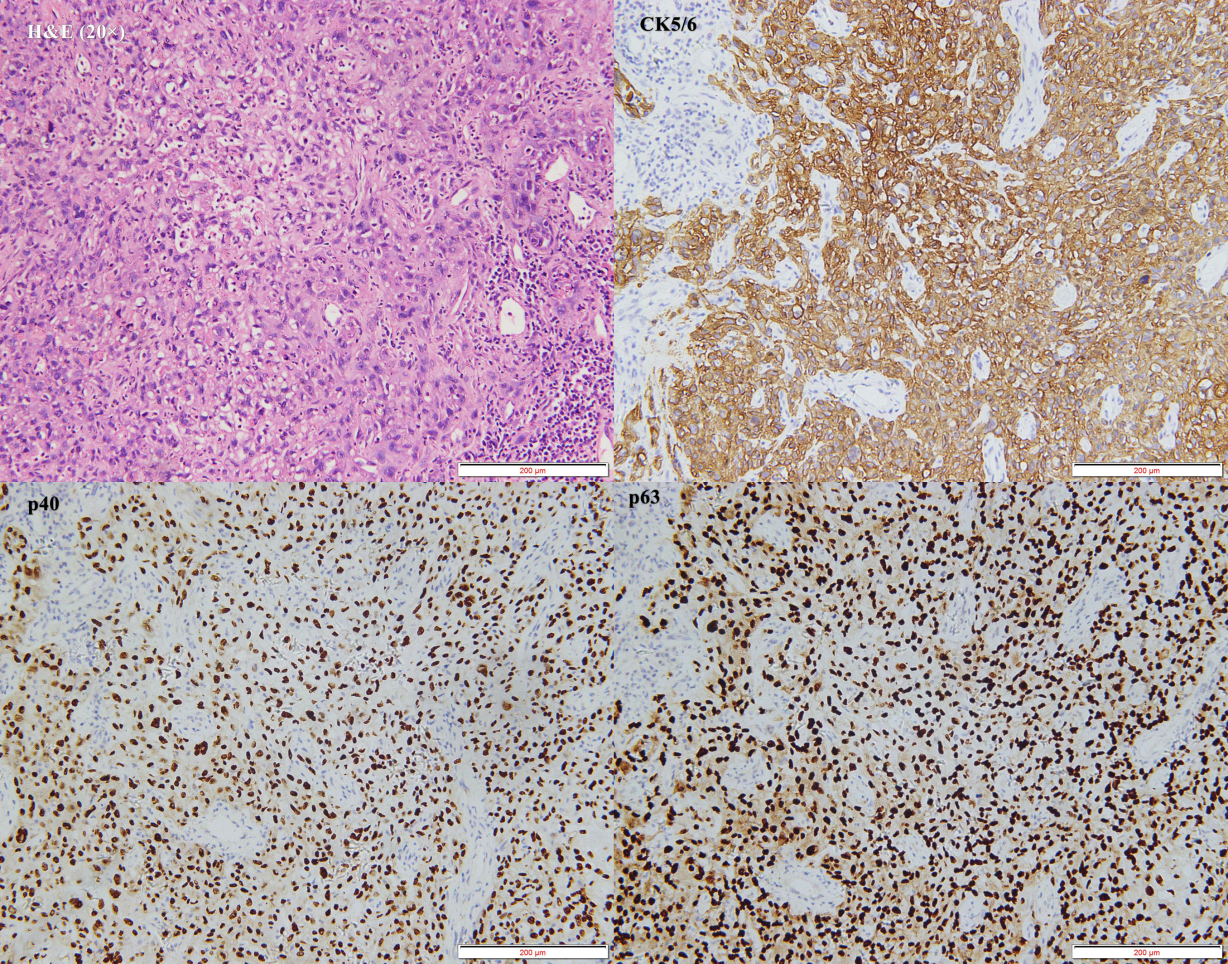
Pathological findings following biopsy.

The patient was initially diagnosed with a hypopharyngeal neoplasm (suspected hypopharyngeal carcinoma), laryngeal obstruction (II°), and bilateral vocal cord fixation. A tracheotomy was performed, and dyspnea was relieved. A computed tomography (CT) scan of the neck on April 27, 2023, revealed a large mass in the hypopharynx and cervical esophagus, with the larynx, trachea, thyroid, and prevertebral fascia involved (**Figure 3**). On April 28, 2023, the patient underwent a biopsy of the hypopharyngeal lesion under general anesthesia. The postoperative pathological results confirmed keratinizing squamous cell carcinoma (moderately differentiated) (**Figure 4**). Immunohistochemical results revealed CAM5.2 (partial+), CK5/6 (+), p40 (+), p63 (+), EGFR (95% membrane strength +), p53 (5% +), and Ki-67 (90% +) expression. According to the American Joint Commission on Cancer (AJCC) eighth edition TNM staging system, the patient was diagnosed with hypopharyngeal and esophageal squamous cell carcinoma (cT4bN0M0, stage IV B). After multidisciplinary team (MDT) consultation, the patient was determined to have surgical indications; however, one-stage surgical resection can result in substantial trauma, and preserving laryngeal function is difficult. The patient also experienced severe depression and refused any form of invasive treatment, such as surgery or radiotherapy, limiting the choice of subsequent treatment and introducing uncertainty regarding the treatment effect. Finally, the patient agreed to use single-mode immunotherapy combination chemotherapy for treatment and a written informed consent form was obtained.

**Figure 5:**
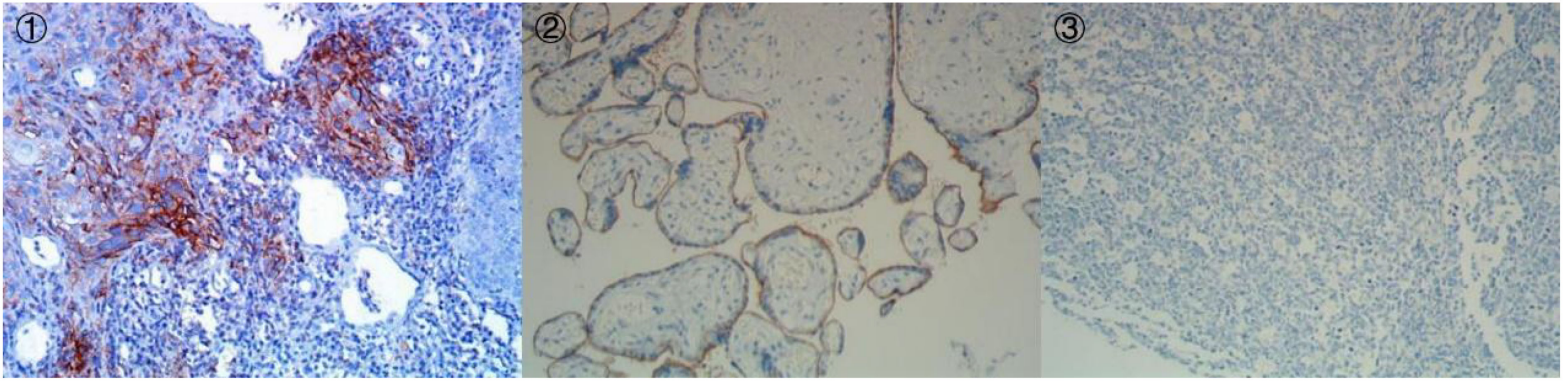
Immunohistochemistry of PD-L1 (IHC, DAKO 22C3) (① PD-L122C3 200×;② PD-L122C3 Positive Control 200×;③ PD-L122C3 Negative Control 200×).

On May 4, 2023, the patient began the first course of neoadjuvant chemotherapy, which included paclitaxel-albumin (80 mg/m^2^), and carboplatin (AUC=2), combined with cetuximab (initial dose, 400 mg/m^2^; subsequent dose, 250 mg/m^2^) (PCC), and continued the next course of treatment every week. After the end of the first treatment cycle, the patient could tolerate tracheal cannula blockage and mild physical activity. On May 8, 2023, tumor biopsy samples from patients were subjected to whole-exome sequencing (WES), and the results revealed that the expression level of programmed death receptor ligand (PD-L1) protein was TPS (Tumor Proportion Score) = 10%, CPS (Combined Positive Score) = 10 (Detection Atlas see **Figure 5**). The following gene mutations were detected: RAD50 (26.88%), MLH1 (23.59%), TP53 (36.34%), and PIK3CA (10.21%). The patient began receiving 200 mg pembrolizumab every 3 weeks from the 3^rd^ course of PCC treatment.

After 7 courses of PCC treatment, electronic laryngoscopy revealed that the hypopharyngeal tumor had nearly disappeared, and the movement of the left vocal cord had returned to normal (**Figure 2**). After 12 cycles of PCC treatment, a CT scan of the neck revealed that the tumor in the hypopharynx and cervical esophagus had significantly decreased. The patient was determined to have achieved CR (**Figure 3**). After 18 cycles of PCC treatment (200 mg of pembrolizumab every 3 weeks), the patient continued to use pembrolizumab combined with cetuximab for maintenance therapy. After 21 months of follow-up, the tumor was in continuous remission, and there was no tumor recurrence. The tracheal cannula was removed, and the patient’s voice, swallowing, and respiratory conditions returned to normal. The mental state and physical condition significantly improved, and the body weight and nutritional status significantly improved (KPS = 100). In addition, the main adverse events occurred during treatment in this patients included mild nausea, vomiting and grade 1–2 immunotherapy-related rash.

## Discussion

The treatment of locally advanced hypopharyngeal carcinoma is difficult, and the prognosis is poor. The difficulty in treating these patients lies in reducing the tumor burden and decreasing the risk of distant metastasis in the short term. Direct surgery can reduce the tumor burden in a short time; however, achieving organ function preservation is traumatic and difficult. The treatment effect of concurrent chemoradiation is unclear; once the cancer relapses, salvage treatment in the later stage may lead to major complications, and radiotherapy can also cause substantial long-term damage to laryngeal function. We reported for the first time that after neoadjuvant immunotherapy combined with chemotherapy resulted in CR in this case of locally advanced hypopharyngeal carcinoma, the continued use of immunotherapy combined with cetuximab for maintenance, without surgery or radiotherapy, achieved continuous CR. Moreover, the patient’s function was well preserved, and the side effects associated with radiotherapy were avoided. These findings suggest that single-mode neoadjuvant immunochemotherapy can reduce the tumor burden, improve local control, and decrease distant metastasis in LA-HNSCC patients. Moreover, it can prolong the survival of patients and preserve their laryngeal function. Furthermore, this single-mode immunochemotherapy is feasible for some LA-HNSCC patients and may be an important supplementary treatment to radiotherapy and surgery. However, the selection of patients and screening of indicators that can predict the efficacy of immunotherapy are the keys to determining patient prognosis. Prospective clinical studies are required to verify our results.

Neoadjuvant chemotherapy can reduce the tumor volume, eliminate micrometastasis, and decrease the local recurrence rate. It is effective for treating LA-HNSCC, among which the TPF (Paclitaxel+Platinum+Fluorouracil) regimen is the classical approach (8–10). However, related studies have shown that although TPF neoadjuvant chemotherapy can reduce staging and metastasis in LA-HNSCC patients, the overall survival benefit is insignificant compared with that achieved with direct surgery (without neoadjuvant chemotherapy) (11, 12).

Immunotherapy has become the first-line treatment for R/M-HNSCC. In 2019, a global multicenter phase III clinical study (KEYNOTE-048) compared immunotherapy related to pembrolizumab with classic EXTREME regimens. The results revealed that OS rates in R/M-HNSCC patients were much greater than those in patients treated with cetuximab combined with platinum chemotherapy. The survival benefit is more obvious in patients with a CPS≥20. This finding also prompted pembrolizumab combined with platinum chemotherapy as a new standard first-line treatment for patients with R/M-HNSCC (13). During the 4-year follow-up, the combination of first-line pembrolizumab with platinum chemotherapy continued to result in better survival benefits in R/M-HNSCC patients than the EXTREME regimen did (14).

An increasing number of studies have focused on neoadjuvant immunotherapy for LA-HNSCC, especially preoperative neoadjuvant immunotherapy. A phase II clinical trial (NCT02641093) conducted by T. M. Wise-Draper et al. on 92 patients with resectable LA-HNSCC revealed that, as a neoadjuvant or adjuvant immunotherapy, pembrolizumab can prolong the one-year tumor-free survival (DFS) of moderate-risk HNSCC patients with negative margins (15). The 1-year DFS rate of patients with a pathological response after neoadjuvant therapy was also significantly higher than that of patients without a pathological response. These findings suggest that the pathological response is positively correlated with survival benefits. A randomized, controlled, open-label phase II clinical trial (NCT05522985) of neoadjuvant immunotherapy (paclitaxel plus cisplatin) for the treatment of resectable locally advanced HNSCC conducted by H. Wang et al. revealed that, compared with induction chemotherapy alone, neoadjuvant chemotherapy combined with immunotherapy could significantly improve the MPR rate and pathological tumor remission (pTR) rate of HNSCC patients. The related adverse events are generally tolerable (16). Gong et al. conducted a phase II clinical trial (NCT04156698) to determine the efficacy and safety of induction chemoimmunotherapy with camrelizumab plus modified TPF in locally advanced hypopharyngeal squamous cell carcinoma, and the results showed a high objective response rate (ORR) with an acceptable safety profile (17). In 2024, a single-center, single-arm pilot phase II trial (NCT04826679) reported by Wu et al. evaluated the efficacy and safety of neoadjuvant chemoimmunotherapy with camrelizumab plus nab-paclitaxel and cisplatin (NeoCPC) in patients with LA-HNSCC. The results showed high remission rates (ORR=89.6%) and controllable safety, which may provide new strategies for organ preservation in LA-HNSCC. In the future, large-scale Phase III trials are needed to verify long-term survival benefits and explore individualized adjustments to therapeutic effects, such as surgical scope, adjuvant treatment intensity (7).

The results above suggest that monoimmunotherapy or combined chemotherapy as a neoadjuvant therapy in LA-HNSCC patients is safe and feasible and that patients who achieve pCR after neoadjuvant immunotherapy tend to have a better prognosis. However, after patients achieve CR or pCR who received neoadjuvant immunotherapy, can they continue immunotherapy for maintenance (surgery and radiotherapy as salvage methods)? This problem is worthy of discussion and in-depth study. In this case, hypopharyngeal and esophageal tumors were significantly reduced after 3 months of neoadjuvant immunochemotherapy. A CT scan of the neck revealed that the hypopharyngeal and esophageal tumors achieved CR. According to the traditional NCCN standard, if the curative effect on the primary tumor is evaluated as CR after neoadjuvant chemotherapy, further radical radiotherapy/chemotherapy or surgery is recommended. However, how can subsequent treatment be chosen for patients who achieve CR after neoadjuvant immunochemotherapy? There is no unified recommendation in the current guidelines. Given the potential long-term benefits of immunotherapy once it begins to benefit, can immunotherapy continue to be maintained for this patient, with surgery or radiotherapy as a salvage treatment? Although we strongly recommended surgery or radiation as radical treatment, this patient explicitly refused any invasive treatment, including radiotherapy and surgery. However, this approach provided a natural observation window for our question: after patients who receive neoadjuvant immunochemotherapy achieve CR, can long-term survival be achieved with immunotherapy for maintenance, without surgery or radiotherapy? The results of the patient’s follow-up have provided an affirmative answer. Our results suggested that for some selected LA-HNSCC patients who cannot preserve their larynx after primary surgery and whose curative radiotherapy effect is questionable, continuing to use immunotherapy to maintain after achieving CR with neoadjuvant immunotherapy may be a good supplement to current treatment methods.

Given that only one-third of patients can benefit from the persistent remission of immune checkpoint inhibitors (ICIs), further exploration of more accurate indicators that can predict the effect of immunotherapy is important. We conducted further whole-exome sequencing (WES) in this case and found mutations in RAD50, MLH1, TP53, and PIK3CA to different degrees. Because the CPS = 10 of this patient did not indicate high expression of PD-L1, the above gene mutation profile may be an important indicator of her continuous remission.

The DNA mismatch repair (MMR) protein *MLH1* is a human protein encoded by the *MLH1* gene located on chromosome 3. Many studies have confirmed that *MLH1* gene inactivation is related to the occurrence of a variety of human solid tumors, such as hereditary nonpolyposis colorectal cancer (Lynch syndrome). Other related studies have shown that promoter hypermethylation may be an important mechanism of *MLH1* gene inactivation in HNSCC (18). In addition, the synergistic effects of *FHIT*, *BRCA2*, *MLH1*, and other related factors may be the molecular basis of esophageal cancer (19).

Because genetic susceptibility to Lynch syndrome is a pathogenic variation of one of the four MMR genes (*MLH1*, *MSH2*, *MSH6*, or *PMS2*), most Lynch syndrome patients have MMR deficiency, MSI, and immune response system activation; thus, Lynch syndrome patients may be the best candidates for ICI therapy (20). The analysis of four MMR genes in this patient revealed a frameshift mutation in the *MLH1* gene (mutation abundance was 23.59%), which may partially explain the increased sensitivity to immunotherapy.

TP53 is the most frequently mutated gene among many in human cancer (>50%). Numerous studies have confirmed that TP53 mutation is closely related to lung cancer, breast adenocarcinoma, and pancreatic cancer (21–23). A large number of studies have shown that the TP53 mutation rate in head and neck tumors is greater than 40% (24). Many studies have demonstrated a significant association between TP53 mutation and HNSCC (25, 26), which may be associated with the TMB. Hongli Gong et al. recently completed the phase II clinical trial of induction chemoimmunotherapy for advanced hypopharyngeal cancer based on carrelizumab (R & D code SHR-1210, trade name: Erica^®^). The results showed that TP53 is the most common mutant gene, and high TMB and CD8+T cells may be predictive biomarkers of the curative effect before treatment. However, this observation from a limited number of clinical tumors must be confirmed by further research (17).

The successful implementation of precision medicine is highly dependent on clinically related predictive biomarkers. No other clear immunomodulatory markers have been identified in HNSCC other than PD-L1. Yi-Hui Pan et al. established a TP53/PIK3CA/ATM mutation classifier using the ICI cohort of Memorial Sloan Kettering Cancer Center (MSKCC) and explored the molecular spectrum and immune infiltration characteristics of each subgroup defined by the classifier. The final results showed that the classifier can predict the effective response rate of patients with bladder cancer to ICI treatment and guide clinical ICI treatment decisions according to different risk levels. However, the mechanisms of TP53/PIK3CA/ATM mutation and the responses to ICI treatment are unclear (27). Sacconi et al. used gene expression profiles from a large database of HNSCC patients with good characteristics (TCGA cohort) to evaluate the role of TP53 gene status and codriven mutations as prognostic predictors for classifying HNSCC patients. Their results revealed a significant association between TP53 gene status and OS and PFS in HNSCC patients. Surprisingly, compared with tumors with TP53 gene mutations alone, the presence of TP53 mutation and another codependent mutation was significantly associated with increased immune gene expression levels. The immune score of HNSCC patients with TP53/FAT1, TP53/CDKN2A, and TP53/PIK3CA comutations was greater than that of patients with TP53 mutations alone (26). These findings suggest that there were not only TP53 mutations but also RAD50 and PIK3CA comutations in this patient, which may be important reasons for the benefit of immunotherapy. Large-scale clinical studies are needed to verify the relationship between these gene mutations and the benefits of immunotherapy.

Certain limitations must be addressed. First, This study is a single-case report. The characteristics of the case reporting itself may limit its promotional value. Therefore, Future clinical studies with large samples may be needed to further validate our results; second, the case was of a female patient, unlike our clinically common hypopharyngeal cancers that are all male patients, and had no history of alcohol or tobacco using. It indicates that the pathogenesis and response to treatment may be different from that of general male patients with hypopharyngeal cancer; third, this case differs from the clinical study. Clinical studies are often performed strictly following the established protocol, however, the treatment strategy for this case can be adjusted according to the condition of the patient, including chemotherapy regimen and cycles, immunotherapy maintenance time and so on.

In summary, patients with hypopharyngeal carcinoma involving cervical esophageal invasion treated by single-mode therapy with pembrolizumab plus chemotherapy have the following advantages: (1) the combination of ICIs and chemotherapy has synergistic antitumor effects, which help improve the response rate and even CR rate of the lesion, leading to excellent local control and long-term survival; (2) treatment-related adverse reactions are mild and well tolerated, indicating suitability for patients who cannot tolerate standard chemoradiation; and (3) laryngeal function is well preserved, and the damage to speech and swallowing function caused by surgery or radiotherapy is completely avoided. However, longer-term follow-up is needed to observe the treatment effect and local tumor control capabilities of this single-mode therapy. In general, the successful use of single-mode therapy in our patient provides a new idea for the treatment of locally advanced hypopharyngeal cancer and indicates a potential new treatment strategy. Large-scale clinical trials are needed to verify the effectiveness and safety of this scheme.

## Data Availability

The original contributions presented in the study are included in the article/supplementary materials, further inquiries can be directed to the corresponding author/s.
