# Supplementary material for: Continuous remission of single-mode therapy with pembrolizumab plus chemotherapy in locally advanced hypopharyngeal carcinoma: a case report
Source: Front Immunol. 2025 Jun 5;16:1557565. doi: 10.3389/fimmu.2025.1557565 (PMC12176769; doi:10.3389/fimmu.2025.1557565)
Supplement: Supplementary file 2 [file 35 Genetic Test Report (188 Gene + PD-L1 Test).pdf › Peer Review Only (2)/Pathology Images/35 Genetic Test Report (188 Gene + PD-L1 Test).pdf]

肿瘤个体化用药

188基因+PD-L1检测

基因检测报告

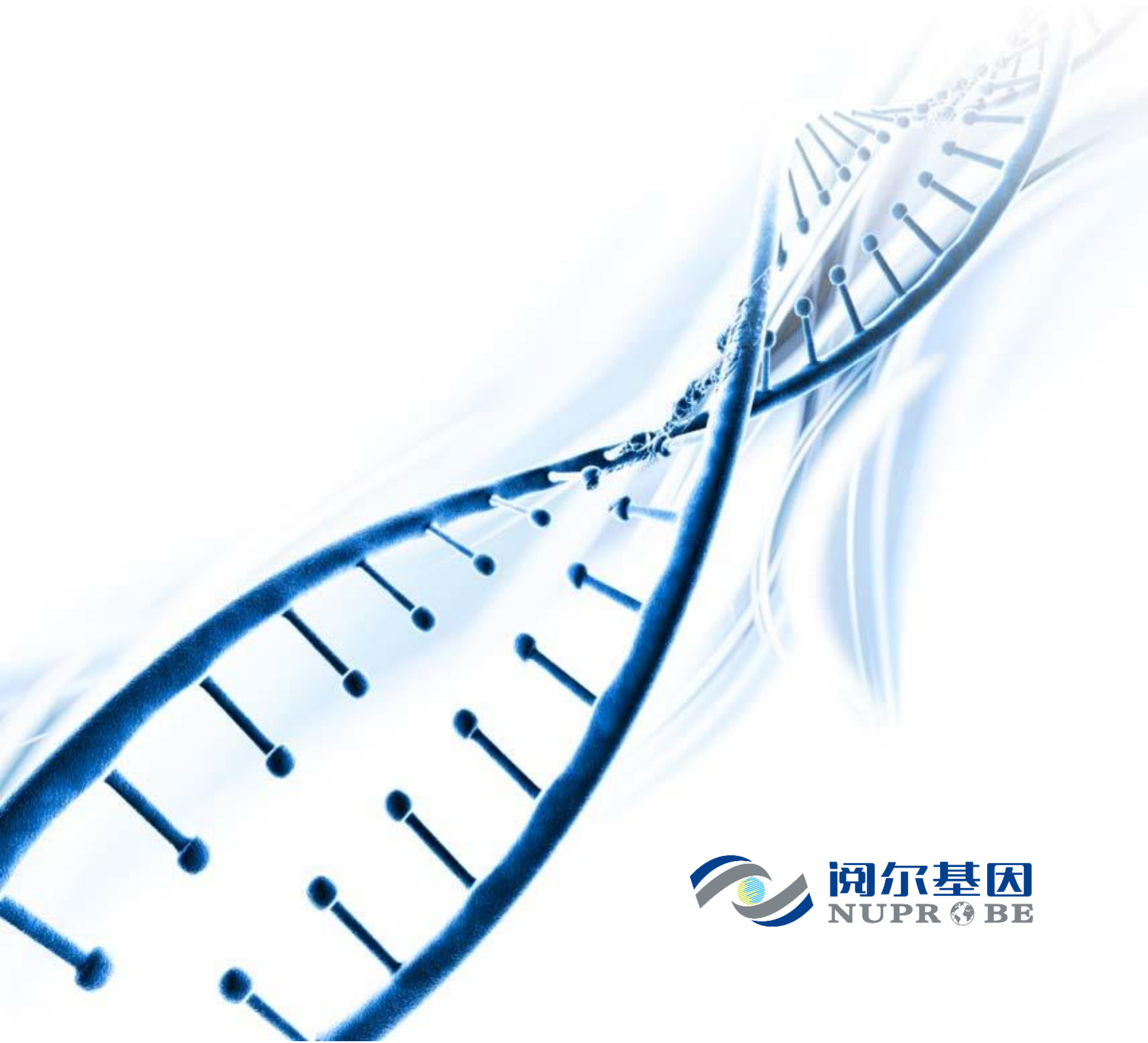

阅尔基因
NUPROBE

Contents 目 录

样本信息......2

检测项目......2

检测结果小结......3

检测结果及用药提示......4

靶向药物用药提示......4

免疫药物用药提示......6

肿瘤遗传风险检测......11

PD-L1 检测结果......12

基因检测结果解析......13

靶向药物检测解析......13

免疫检测结果解析......16

化疗药物检测解析......17

附录......26

样本质控情况......26

常见靶向药物相关基因检测列表......27

检测基因列表......35

检测流程......37

参考文献......38

样本信息

姓名：[redacted]	送检医院：-	样本条码：[redacted]
性别：女	送检科室：-	样本编号：[redacted]
年龄：69	送检医生：-	门诊/住院号：-
联系电话：-	标本类型：组织	送检时间：2023-05-08
临床诊断：下咽癌鳞癌	订单编号：-	接收时间：2023-05-09

检测项目

本产品对与肿瘤密切相关的 188 个基因进行高通量测序。检测突变形式为点突变（SNV）、小片段插入缺失（INDEL）、拷贝数变异（CNV）以及融合（FUSION）。通过免疫组化检测 PD-L1 表达。

本报告分析基因变异与靶向药物、免疫药物、化疗药物和遗传风险的相关性，给出靶向药物（FDA/NMPA 批准药物、临床试验药物等）、化疗药物的用药提示信息，从而为临床制定治疗方案提供参考信息。

注：检测基因列表见附录。

检测结果小结

检测类型		检测结果
靶向用药指导		共检出 6 个体细胞突变，其中 1 个与靶向药物相关（RAD50 p.K722Rfs*14）；<br>共检出 1 个胚系突变，其中 0 个与靶向药物相关
免疫治疗相关标志物检测	微卫星不稳定性（MSI）	MSS
	MMR 基因检测	共检出 0 个 MMR 基因致病/可能致病性胚系突变
肿瘤遗传风险检测		共检出 1 个胚系突变，其中 0 个为致病/可能致病性突变
PD-L1 蛋白表达水平		使用 DAKO 22C3 抗体；TPS: 10%， CPS : 10
化疗药物检测		详见“化疗药物检测解析”部分
样品总体质量评估		合格

说明：

1. 本报告为基因检测结果，基因、药物等信息列举未按照重要性排序。
2. 检测结果仅供科学研究和临床用药参考，具体治疗方案请咨询主治医生。
3. 本报告只对本次采集样本负责，如有疑问，请在 7 个工作日内与我们联系。

检测结果及用药提示

◆ 靶向药物用药提示

本次样本共检出 6 个体细胞突变，其中 1 个与靶向药物相关；共检出 1 个胚系突变，其中 0 个与靶向药物相关。具体信息详见“基因检测结果解析”。

突变基因	检测结果	突变丰度	可能获益的药物			可能耐药药物
			A 级	B 级	C 级	
RAD50	NM_005732.4 exon13 c.2165del p.K722Rfs*14	26.88%	无	无	尼拉帕利 + 卡铂#，帕米帕利 + 替雷利珠单抗#	无

注：

1. 突变丰度：在某位点产生突变的等位基因在该位点全部等位基因中所占比率。
2. 可能获益药物：A 级：FDA/NMPA 已经批准或各指南推荐在本癌种该靶点上可用的药物；B 级：大型已注册 II、III、IV 期临床试验证实在本癌种该靶点可用的药物；C 级：其他癌种 A 级证据，或 I 期临床试验/临床个例/研究者独立开展的多病例小型临床研究证实在本癌种该靶点可用的药物，或本癌种该靶点用药符合正在招募的大型已注册临床试验入组标准。
3. 可能耐药药物：FDA/NMPA 已经批准的、NCCN 指南推荐的、临床试验或者临床研究有明确结果的、药物敏感性可能降低或产生耐药的靶向药物。
4. 黑色粗体药物：FDA/NMPA 已批准药物，未加粗药物为未上市药物。
5. * 标注的药物：NMPA 批准的靶向药物。
6. # 标注的药物：符合正在招募的大型已注册临床试验入组标准的药物。

未知临床意义或无相关靶向用药的基因突变

基因名称	检测结果	突变类型	突变丰度
MLH1	NM_000249.4 exon19 c.2252_2253del p.K751Sfs*3	移码突变	23.59%
TP53	NM_000546.6 exon2 c.46del p.Q16Rfs*28	移码突变	36.34%
PIK3CA	NM_006218.4 exon20 c.2932G>A p.E978K	错义突变	10.21%
BRIP1	NM_032043.3 exon20 c.2915T>C p.V972A	错义突变	10.69%
POLE	NM_006231.4 exon7 c.650A>G p.N217S	错义突变	3.38%

注:

1. 未知临床意义的基因突变是指在出具报告时文献尚未报道，或者是突变意义已经明确但是无相关的靶向用药信息，或者是该突变意义不明确，可能存在潜在的临床意义。

◆ 免疫药物用药提示

微卫星不稳定（MSI）检测结果

检测项目	检测结果
微卫星不稳定（microsatellite instability,**MSI**）	MSS

注:

1. 微卫星不稳定（MSI）是由于在 DNA 复制时插入或缺失突变引起的微卫星序列长度改变的现象。MSI 结果可以分为微卫星稳定（MSS）型和微卫星不稳定（MSI-H）型。MSI-H 型结直肠癌、胃癌、子宫内膜癌、胆管癌及胰腺癌等患者可能从免疫治疗 PD-1 抑制剂类药物中获益。
2. 本次检测使用 NGS 技术结合生物信息学算法，基于内部验证数据，通过微卫星位点的长度分布评估判断 MSI 状态。

错配修复（MMR）相关基因检测结果

基因名称	检测结果	突变丰度	突变类型
MLH1	NM_000249.4 exon19 c.2252_2253del p.K751Sfs*3	23.59%	移码突变
MSH2	未检测到相关基因突变	/	/
MSH6	未检测到相关基因突变	/	/
PMS2	未检测到相关基因突变	/	/

注:

1. MLH1、MSH2、MSH6 和 PMS2 胚系突变常导致林奇综合征，结直肠癌、胃癌、子宫内膜癌等多种癌症的风险增高。同时，多项回顾性研究表明，MMR 基因的体细胞突变同样可以造成 dMMR/MSI-H，与散发性结直肠癌和子宫内膜癌发生相关[PMID: 24333619; PMID: 25194673]。
2. 一项临床研究表明，帕博利珠单抗用于 dMMR/MSI-H 的结直肠癌患者的客观缓解率（ORR）为 36%，非结直肠癌患者的 ORR 为 46%[FDA Reference ID:4389012]。基于该研究，FDA 已批准帕博利珠单抗用于 dMMR/MSI-H 的治疗进展后没有合适的替代治疗方案的儿童或者成年实体瘤患者的治疗；或者氟尿嘧啶、奥沙利铂和伊立替康治疗后进展的结直肠癌患者的治疗。
3. CheckMate 142 的临床研究结果显示，纳武利尤单抗用于 dMMR/MSI-H 的结直肠癌患者的 ORR 为 28%，包括 1 例完全缓解和 14 例部分缓解[PMID: 28734759]。基于该研究，FDA 已批准纳武利尤单抗用于 dMMR/MSI-H 的氟尿嘧啶、奥沙利铂和伊立替康为基础治疗进展后的成人和儿童（12 岁及以上）的晚期结直肠癌患者。
4. 本次检测仅限于基因水平突变检测，未进行 IHC 表达检测，仅供参考。

目前各癌种已批准的免疫治疗药物								
**商品名**	**Opdivo**	**Keytruda**	**Libtayo**	**拓益**	**达伯舒**	**艾瑞卡**	**百泽安**	**Jemperli**
**药物名称**	**Nivolumab**	**Pembrolizumab**	**Cemiplimab-rwlc**	**Toripalimab**	**Sintilimab**	**Camrelizumab**	**Tislelizumab**	**Dostarlimab**
**中文译名（参考）**	**纳武利尤单抗（欧狄沃）**	**帕博利珠单抗（可瑞达）**	**暂无**	**特瑞普利单抗**	**信迪利单抗**	**卡瑞利珠单抗**	**替雷利珠单抗**	**暂无**
**靶点**	**PD-1**	**PD-1**	**PD-1**	**PD-1**	**PD-1**	**PD-1**	**PD-1**	**PD-1**
鼻咽癌	/	/	/	NMPA	/	NMPA	NMPA	/
头颈癌	FDA NMPA	FDA NMPA	/	/	/	/	/	/
非小细胞肺癌	FDA NMPA	FDA NMPA	FDA	NMPA	NMPA	NMPA	NMPA	/
食管癌	FDA NMPA	FDA NMPA	/	NMPA	NMPA	NMPA	NMPA	/
胃食管结合腺癌	FDA NMPA	FDA NMPA	/	/	NMPA	/	/	/
胃癌	FDA NMPA	FDA	/	/	NMPA	/	/	/
结直肠癌	FDA	FDA NMPA	/	/	/	/	/	/
三阴性乳腺癌	/	FDA	/	/	/	/	/	/
宫颈癌	/	FDA	/	/	/	/	/	/
子宫内膜癌	/	FDA	/	/	/	/	/	FDA
胸膜间皮瘤	FDA NMPA	/	/	/	/	/	/	/
肝细胞癌	FDA	FDA NMPA	/	/	NMPA	NMPA	NMPA	/
肾细胞癌	FDA	FDA	/	/	/	/	/	/
尿路上皮癌	FDA NMPA	FDA	/	NMPA	/	/	NMPA	/
黑色素瘤	FDA	FDA NMPA	/	NMPA	/	/	/	/
皮肤鳞状细胞癌	/	FDA	FDA	/	/	/	/	/
默克尔细胞癌	/	FDA	/	/	/	/	/	/
基底细胞癌	/	/	FDA	/	/	/	/	/
实体瘤	/	FDA	/	/	/	/	NMPA	FDA
霍奇金淋巴瘤	FDA	FDA	/	/	NMPA	NMPA	NMPA	/
非霍奇金淋巴瘤	/	FDA	/	/	/	/	/	/

注：表中已获批药物包括单药获批药物和联药获批药物。信息来源：FDA/NMPA 官网、药企官网发布信息。

目前各癌种已批准的免疫治疗药物							
**商品名**	**誉妥**	**汉斯状**	**开坦尼**	**普佑恒**	**安尼可**	**IMJUDO**	**Yervoy**
**药物名称**	**Zimberelimab**	**Serplulimab**	**Cadonilimab**	**Pucotenlimab**	**Penpulimab**	**Tremelimumab**	**Ipilimumab**
**中文译名（参考）**	**赛帕利单抗**	**斯鲁利单抗**	**卡度尼利单抗**	**普特利单抗**	**派安普利单抗**	**暂无**	**伊匹木单抗**
**靶点**	**PD-1**	**PD-1**	**PD-1**	**PD-1**	**PD-1**	**CTLA-4**	**CTLA-4**
鼻咽癌	/	/	/	/	/	/	/
头颈癌	/	/	/	/	/	/	/
非小细胞肺癌	/	NMPA	/	/	NMPA	FDA	FDA
小细胞肺癌	/	NMPA	/	/	/	/	/
食管癌	/	/	/	/	/	/	FDA
胃食管结合腺癌	/	/	/	/	/	/	/
胃癌	/	/	/	/	/	/	/
结直肠癌	/	/	/	/	/	/	FDA
三阴性乳腺癌	/	/	/	/	/	/	/
宫颈癌	/	/	NMPA	/	/	/	
子宫内膜癌	/	/	/	/	/	/	/
胸膜间皮瘤	/	/	/	/	/	/	FDA<br>NMPA
肝细胞癌	/	/	/	/	/	FDA	FDA
肾细胞癌	/	/	/	/	/	/	FDA
尿路上皮癌	/	/	/	/	/	/	/
黑色素瘤	/	/	/	NMPA	/	/	FDA
皮肤鳞状细胞癌	/	/	/	/	/	/	/
默克尔细胞癌	/	/	/	/	/	/	/
基底细胞癌	/	/	/	/	/	/	/
实体瘤	/	NMPA	/	NMPA	/	/	/
霍奇金淋巴瘤	NMPA	/	/	/	NMPA	/	/
非霍奇金淋巴瘤	/	/	/	/	/	/	/

注：表中已获批药物包括单药获批药物和联药获批药物。信息来源：FDA/NMPA 官网、药企官网发布信息。

目前各癌种已批准的免疫治疗药物						
**商品名**	**Tecentriq**	**Bavencio**	**Imfinzi**	**择捷美**	**恩维达**	**Opdualag**
**药物名称**	**Atezolizumab**	**Avelumab**	**Durvalumab**	**Sugemalimab**	**Envafolimab**	**Nivolumab and Relatlimab**
**中文译名（参考）**	**阿替利珠单抗**	**暂无**	**度伐利尤单抗**	**舒格利单抗**	**恩沃利单抗**	**暂无**
**靶点**	**PD-L1**	**PD-L1**	**PD-L1**	**PD-L1**	**PD-L1**	**PD-1, LAG-3**
非小细胞肺癌	FDA<br>NMPA	/	FDA<br>NMPA	NMPA	/	/
小细胞肺癌	FDA<br>NMPA	/	FDA<br>NMPA	/	/	/
肝细胞癌	FDA<br>NMPA	/	FDA	/	/	/
肾细胞癌	/	FDA	/	/	/	/
尿路上皮癌	FDA	FDA	FDA	/	/	/
黑色素瘤	FDA	/	/	/	/	FDA
默克尔细胞癌	/	FDA	/	/	/	/
实体瘤	/	/	/	/	NMPA	/
腺泡状软组织肉瘤	FDA	/	/	/	/	/
胆道癌	/	/	FDA	/	/	/

注：表中已获批药物包括单药获批药物和联药获批药物。信息来源：FDA/NMPA 官网、药企官网发布信息。

◆ 肿瘤遗传风险检测

共检出 1 个胚系突变，其中 0 个为致病/可能致病性突变。如果您有家族遗传史，可联系我们做进一步的检测。

基因	染色体	外显子	核苷酸	氨基酸	杂合/纯合	突变类型	千人频率	临床意义
BRCA1	17	intron13	c.4484+14A>G	.	杂合	.	0.22%	良性变异

注：

1. 本报告只给出千人频率均在 2%以下的胚系非同义突变。
2. 根据美国医学遗传医师学会（ACMG）指导意见，个体携带的变异，分为 5 个大类：

- 致病性变异：有大量证据表明这种变异是高风险的致病性变异。
- 可能致病性变异：有一些证据表明这种变异是高风险的致病性变异。
- 不确定性变异：没有足够的证据，将变异分类到其他四类中。
- 可能良性变异：有一些证据表明这种变异不是高风险的致病变异。
- 良性变异：有大量证据表明这种变异不是高风险的致病变异。

◆ PD-L1 检测结果

组织切片质控：

肿瘤细胞数是否大于 100 个	是
镜下描述：瘤细胞呈巢状排列，浸润性生长，瘤巢形态不规则，周边间质见促纤维结缔组织增生，伴灶性坏死，瘤细胞呈短梭形，胞浆粉染，细胞核大空泡状，胞浆量少，核浆比明显增大，可见病理性核分裂。	

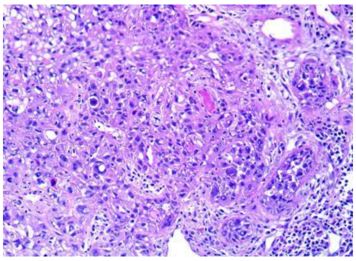

HE 染色切片 200X

检测结果：

检测项目	检测方法	检测抗体	检测结果
PD-L1-22C3	IHC	DAKO 22C3	TPS：10%
			CPS：10

检测图谱：

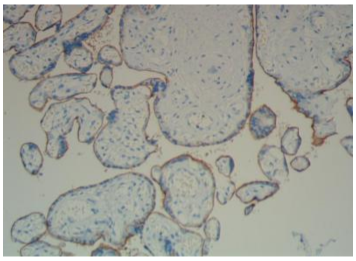

PD-L122C3 阳性对照 200X

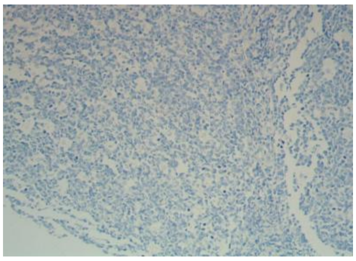

PD-L122C3 阴性对照 200X

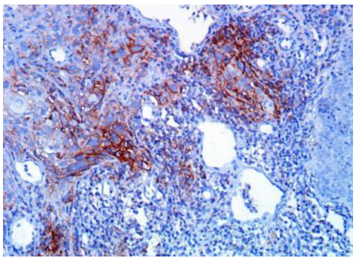

PD-L122C3 200X

注

$$\text{TPS（Tumor Proportion Score）} = \frac{\text{PD-L1 染色细胞(肿瘤细胞)数量}}{\text{活的肿瘤细胞总数}} \times 100\%$$

$$\text{CPS（Combined Positive Score）} = \frac{\text{PD-L1 染色细胞(肿瘤细胞+淋巴细胞+巨噬细胞)数量}}{\text{活的肿瘤细胞总数}} \times 100$$

CPS 结果采用 0~100 数值来表示，当计算结果超过 100 时，最终结果按照 100 来计算。

程序性死亡受体 1(PD-1)是一种重要的免疫抑制分子，主要在激活的 T 细胞和 B 细胞中表达。肿瘤细胞中高表达程序性死亡配体 1(PD-L1)，与 PD-1 结合后可抑制 T 细胞激活，从而使肿瘤细胞躲避免疫系统的识别与攻击。通过 PD-1/PD-L1 抑制剂阻断该信号通路，阻断负向调控信号，能够使 T 细胞恢复活性，增强免疫应答。近期研究发现 PD-1 和 PD-L1 抑制剂在非小细胞肺癌、黑色素瘤、膀胱癌等多种肿瘤中疗效显著,其中肿瘤组织中 PD-L1 的表达水平对预测 PD-1 和 PD-L1 抑制剂疗效具有重要意义。

基因检测结果解析

◆ 靶向药物检测解析

RAD50

突变形式		NM_005732.4 exon13 c.2165del p.K722Rfs*14
变异丰度		26.88%
潜在获益药物	A 级	无
	B 级	无
	C 级	尼拉帕利 ＋ 卡铂#，帕米帕利 ＋ 替雷利珠单抗#
潜在耐药信息		无
基因突变相关说明		**基因说明:**RAD50 基因编码的蛋白与参与 DNA 蛋白双链修复的酿酒酵母蛋白 RAD50 高度相似。该蛋白与 MRE11 及 NBS1 形成复合体后具有多种酶活性，是 DNA 非同源末端连接的必需因子，在 DNA 双链断裂修复、细胞周期检查点的激活、端粒维护、减数分裂重组等过程中具有重要作用。RAD50 发生突变就会阻碍 DNA 重组的发生，或干扰 DNA 的修复系统，造成胚胎的死亡或者癌变。与 RAD50 相关的疾病包括子宫内膜癌、肠癌、胃癌等。<br>**突变说明:**位于 13 号外显子上的第 2165 位缺失核苷酸，导致相应蛋白序列中第 722 位氨基酸赖氨酸(K)被氨基酸精氨酸(R)替代并发生移码，从此位置开始第 14 位为终止密码子(*)，此突变在样本中的突变丰度为 26.88%。该变异为移码突变，可能造成蛋白功能丧失。

临床试验信息

ID	临床试验名称	肿瘤类型	阶段	药物	地点
NCT04985721	一项帕米帕利联合替雷利珠单抗治疗具有同源重组修复缺陷的晚期肿瘤的开放标签、信号寻找、转译的 II 期临床试验	实体瘤	II 期	帕米帕利 ＋ 替雷利珠单抗 (BGB290 + BGB-A317)	澳大利亚
NCT03209401	一项尼拉帕利联合卡铂治疗同源重组缺陷型晚期恶性肿瘤患者的 I 期临床试验	实体瘤	I 期	尼拉帕利 ＋ 卡铂 (MK4827 + CBDCA)	美国

未知临床意义或无相关靶向用药的基因突变解析

MLH1

突变形式	NM_000249.4 exon19 c.2252_2253del p.K751Sfs*3
突变说明	位于 19 号外显子上的第 2252 位到第 2253 位缺失核苷酸，导致相应蛋白序列中第 751 位氨基酸赖氨酸(K)被氨基酸丝氨酸(S)替代并发生移码，从此位置开始第 3 位为终止密码子(*)，此突变在样本中的突变丰度为 23.59%。
基因说明	MLH1 是一种肿瘤抑制因子，其与 PMS2 二聚化形成 DNA 错配修复系统的组分[PMID: 16873062]，同时也参与了 DNA 损伤修复。此外，它与微卫星不稳定性（MSI）相关[PMID: 30121009]。与 MLH1 相关的疾病包括结直肠癌、子宫内膜癌等。

TP53

突变形式	NM_000546.6 exon2 c.46del p.Q16Rfs*28
突变说明	位于 2 号外显子上的第 46 位缺失核苷酸，导致相应蛋白序列中第 16 位氨基酸谷氨酰胺(Q)被氨基酸精氨酸(R)替代并发生移码，从此位置开始第 28 位为终止密码子(*)，此突变在样本中的突变丰度为 36.34%。
基因说明	TP53 是抑癌基因，编码一个包括转录激活、DNA 结合及寡聚化结构域肿瘤抑制蛋白。该编码蛋白能响应不同细胞应激从而调控目标基因表达，进而诱导细胞周期停滞、细胞凋亡、衰老、DNA 修复和代谢变化。该基因突变与多种人类癌症有关，与 TP53 相关的疾病包括软组织和骨肉瘤、乳腺癌、脑癌、肾上腺皮质癌等。

PIK3CA

突变形式	NM_006218.4 exon20 c.2932G>A p.E978K
突变说明	位于 20 号外显子上的第 2932 位核苷酸 G 被核苷酸 A 替代，导致相应蛋白序列中第 978 位氨基酸谷氨酸(E)被氨基酸赖氨酸(K)替代，此突变在样本中的突变丰度为 10.21%。
基因说明	磷脂酰肌醇 3-激酶由一个 85kDa 的调节亚基和一个 110kDa 的催化亚基组成。PIK3CA 编码的 p110-alpha 蛋白是磷脂酰肌醇 3-激酶 (PI3K) 的催化亚基，以 ATP 依赖的方式磷酸化 PtdIns、PtdIns4P 和 PtdIns(4,5)P2。该基因与宫颈癌的发生有关。其所在通路—PI3K/AKT/mTOR 通路可调控细胞的生长、增殖、分化、活力和存活。

BRIP1

突变形式	NM_032043.3 exon20 c.2915T>C p.V972A
突变说明	位于 20 号外显子上的第 2915 位核苷酸 T 被核苷酸 C 替代，导致相应蛋白序列中第 972 位氨基酸缬氨酸(V)被氨基酸丙氨酸(A)替代，此突变在样本中的突变丰度为 10.69%。
基因说明	BRIP1 基因编码的蛋白为 RecQDEAH 解旋酶家族成员之一，在乳腺癌 1 型中，与 BRCT 重复序列相互作用，该络合物在 DNA 双链修复中具有重要作用。该基因属于抑癌基因，若发生突变则会引起 DNA 紊乱从而诱导癌症的发生。与 BRIP1 相关的疾病包括乳腺癌、子宫内膜癌、肠癌、胃癌等。

POLE

突变形式	NM_006231.4 exon7 c.650A>G p.N217S
突变说明	位于 7 号外显子上的第 650 位核苷酸 A 被核苷酸 G 替代，导致相应蛋白序列中第 217 位氨基酸天冬酰胺(N)被氨基酸丝氨酸(S)替代，此突变在样本中的突变丰度为 3.38%。
基因说明	POLE 基因全长 63.5kb，位于 12q24.3。该基因编码人类 DNA 聚合酶ε的 285kD 催化活性亚基，具有

	DNA 多聚酶活性和 3′外切活性，在真核生物中高度保守，主要功能为 DNA 的复制和损伤修复。与 POLE 相关的疾病包括肺癌、子宫内膜癌、结直肠癌、胃癌等。

◆ 免疫检测结果解析

微卫星不稳定（MSI）检测结果解析

**检测项目**	微卫星不稳定（microsatellite instability,**MSI**）
**检测结果**	MSS
**基因突变相关说明**	**MSI 定义:**指 DNA 序列中简单重复序列的碱基长度和（或）重复次数的增加或减少，产生遗传不稳定性。<br>**位点说明:**MSS 代表微卫星稳定。

◆ 化疗药物检测解析

化疗药物毒副作用风险解析

化疗药物	检测基因	检测位点	检测结果	毒副作用风险用药提示(仅供参考)	等级
阿那曲唑	ABCB1	rs1045642	GG	携带 GG 基因型的乳腺癌患者，相比于 AA 基因型，在接受阿那曲唑治疗时可能更容易出现关节痛。[PMID:27747906]	3
阿那曲唑 + 依西美坦	MAP4K4	rs4550690	TT	携带 TT 基因型的乳腺癌患者，与 CT 或 CC 基因型患者相比，暴露于芳香化酶抑制剂阿那曲唑和依西美坦时，发生骨折的风险可能会增加。[PMID:25148458]	3
奥沙利铂	GSTP1	rs1695	AG	携带 AG 基因型的结直肠癌患者，相比于 GG 基因型，使用奥沙利铂治疗时神经毒性综合征的严重程度降低相关。[PMID:17401013]	3
奥沙利铂	GSTP1	rs1695	AG	携带 AG 基因型的胃肠道癌患者，相比于 AA 基因型，使用奥沙利铂治疗周围神经系统疾病的严重程度降低相关。[PMID:16707601]	3
铂类	ERCC2	rs1799793	CC	携带 CC 基因型非小细胞肺癌患者在接受铂基化疗时，与 TT 基因型相比，可能有 1)肺炎风险降低，2)与药物毒性相关的不良事件风险增加。[PMID:25069034, PMID:27498158]	3
铂类	ERCC1	rs11615	GG	携带 GG 基因型的非小细胞肺癌患者，与 AA 或 AG 基因型相比，在使用铂类治疗时可能会减少毒性风险。[PMID:27498158]	3
铂类	SLC22A2	rs316019	CC	携带 CC 基因型的非小细胞肺癌患者，与 AA 和 AC 基因型相比，在使用铂类治疗时，可能会降低毒性的严重程度，特别是肝毒性。[PMID:27590272]	3
铂类	ABCB1	rs1128503	GG	携带 GG 基因型的非小细胞肺癌患者，与 AA 基因型患者相比，在接受铂基化疗治疗时其毒性风险可能降低。[PMID:27498158]	3
铂类	GSTP1	rs1695	AG	携带 AG 基因型的非小细胞肺癌患者，相比于 AA 基因型，使用铂类药物治疗时中性粒细胞减弱的风险降低相关。[PMID:17409936]	3
铂类	GSTP1	rs1695	AG	携带 AG 基因型的癌症患者，相比于 AA 基因型，在接受铂类药物治疗时可能具有更低的毒性风险。[PMID:15213713, PMID:17409936, PMID:27995989]	3
铂类	ERCC2	rs13181	TT	携带 TT 基因的非小细胞肺癌患者，与 GG 基因型相比，在使用铂类治疗时患肺炎的风险可能降低。[PMID:25069034]	3
博来霉素 + 顺铂 + 依托泊苷	ERCC1	rs11615	GG	携带 GG 基因的睾丸癌患者，与 AA 基因型相比，在使用博来霉素、顺铂和依托泊苷治疗后发生发热性中性粒细胞减少症的风险可能降低。[PMID:30914949]	3
博来霉素 + 顺铂 + 依托泊苷	ERCC1	rs3212986	CC	携带 CC 基因型的睾丸癌患者，相比于 AA 基因型，在使用博来霉素、顺铂和依托泊苷治疗后，呕吐风险可能降低。[PMID:30914949]	3
博来霉素 + 顺铂 + 依托泊苷	ERCC2	rs1799793	CC	携带 CC 基因型睾丸癌患者，与 TT 基因型相比，在接受博来霉素、顺铂和依托泊苷治疗后发生贫血的风险可能降低。[PMID:30914949]	3
博来霉素 + 顺铂 + 依托泊苷	GSTP1	rs1695	AG	携带 AG 基因型的睾丸癌患者，相比于 GG 基因型，在接受博来霉素、顺铂和依托泊苷治疗后，感染或恶心的风险可能降低。[PMID:30914949]	3
博来霉素 + 顺铂 + 依托泊苷	ERCC2	rs13181	TT	携带 TT 基因型的睾丸癌患者，与 GG 或 GT 基因型相比，在使用博来霉素、顺铂和依托泊苷治疗时而发生脱发的风险可能降低。[PMID:30914949]	3
博来霉素 + 顺铂 + 依托泊苷	ABCB1	rs1045642	GG	携带 GG 基因型的睾丸癌患者，相比于 AA 基因型，使用博来霉素、顺铂和依托泊苷治疗后，呕吐的风险可能会降低。[PMID:30914949]	3
多柔比星	GSTP1	rs1695	AG	携带 AG 基因型的骨肉瘤患者，相比于 AA 基因型，在接受多柔比星治疗时发生白细胞减弱的风险可能更高。[PMID:21887680]	3
多西他赛	ERCC1	rs11615	GG	携带 GG 基因型的乳腺癌患者，与 AA 基因型相比，在使用多西他赛治疗时发生粘膜炎的风	3

化疗药物	检测基因	检测位点	检测结果	毒副作用风险用药提示(仅供参考)	等级
				险可能降低。[PMID:25495407]	
多西他赛	ERCC1	rs3212986	CC	携带 CC 基因的乳腺癌患者，与 AA 或 AC 基因型相比，在使用多西他赛治疗时发生粘膜炎的风险可能增加。[PMID:25495407]	3
氟尿嘧啶	GSTP1	rs1695	AG	携带 AG 基因型的直肠癌患者，相比于 AA 基因型，接受氟尿嘧啶治疗的血液毒性风险可能较低。[PMID:18540691]	3
氟尿嘧啶 + 亚叶酸 + 奥沙利铂	XRCC1	rs25487	CT	携带 CT 基因型结肠癌患者，与 CC 基因型相比，在接受氟尿嘧啶、亚叶酸和奥沙利铂治疗时，发生周围神经系统疾病的风险增加，感觉神经病变的发病时间提前。[PMID:23314736]	3
氟尿嘧啶 + 亚叶酸 + 奥沙利铂	ERCC2	rs13181	TT	携带 TT 基因型的结直肠癌患者，与 GG 基因型相比，在使用氟尿嘧啶、亚叶酸和奥沙利铂治疗时，可能 1）药物毒性风险降低；2）无进展生存期增加。[PMID:20078613, PMID:20385995]	3
氟尿嘧啶 + 亚叶酸 + 奥沙利铂	MTHFR	rs1801133	AG	携带 AG 和 GG 基因型结肠癌患者，与 AA 基因型相比，在接受氟尿嘧啶、亚叶酸和奥沙利铂治疗时，中性粒细胞减少的风险降低。[PMID:23314736]	3
氟尿嘧啶 + 亚叶酸 + 奥沙利铂	ERCC1	rs11615	GG	携带 GG 基因型的结肠癌患者，与 AA 基因型相比，在使用 FOLFOX（氟尿嘧啶、亚叶酸、奥沙利铂）治疗时，中性粒细胞减少的风险可能降低。[PMID:23314736]	3
氟尿嘧啶 + 伊立替康 + 奥沙利铂	GSTP1	rs1695	AG	携带 AG 基因型的结直肠癌患者，相比于 AA 基因型，使用氟尿嘧啶、伊立替康或奥沙利铂治疗时神经毒性综合征和中性粒细胞减弱的风险降低相关。[PMID:20530282]	3
氟尿嘧啶类	DPYD	rs3918290	CC	携带 CC 基因型的癌症患者，相比于 TT 或 CT 基因型，接受氟嘧啶类药物治疗可能降低药物毒性的风险和严重程度。然而，报道的证据相互矛盾。[PMID:25381393, PMID:26099996, PMID:30114658]	1A
氟尿嘧啶类	DPYD	rs55886062	AA	携带 AA 基因型癌症患者，与 AC 或 CC 基因型患者相比，接受氟尿嘧啶类药物治疗，毒性风险有可能降低。[PMID:31745289, PMID:17121937, PMID:26603945]	1A
氟尿嘧啶类	DPYD	rs67376798	TT	携带 TT 基因型的肿瘤患者，与 AT 或 AA 基因型患者相比，在使用氟尿嘧啶类治疗时的药物毒性风险可能降低。[PMID:31745289, PMID:21412232, PMID:23930673]	1A
格拉司琼	ERCC1	rs3212986	CC	携带 CC 基因的肿瘤患者，与 AA 基因型相比，在使用格拉司琼或帕洛诺司琼治疗时在顺铂给药后的前 24 小时内可能有更好的反应和呕吐减少。[PMID:29177570]	3
环磷酰胺	ABCC4	rs9561778	GT	携带 GT 基因型的乳腺癌患者，相比于 GG 基因型，使用环磷酰胺治疗可能会增加药物不良反应的风险，特别是中性粒细胞减少/白细胞减少和胃肠道毒性。[PMID:19696793]	3
环磷酰胺 + 表柔比星	GSTP1	rs1695	AG	-	-
环磷酰胺 + 表柔比星	GSTP1	rs1695	AG	携带 AG 基因型的乳腺癌患者，相比于 GG 基因型，在使用环磷酰胺和表柔比星治疗时，毒性的严重程度可能会降低，反应可能更高。[PMID:21362365]	3
环磷酰胺 + 表柔比星 + 紫杉醇	CYP1B1	rs1056836	GG	携带 GG 基因型乳腺癌患者，与 CC 基因型患者相比，在使用环磷酰胺、表柔比星和紫杉醇治疗时发生感觉神经病变的几率可能增加。[PMID:24599932]	3
环磷酰胺 + 多柔比星	CYP2B6	rs3211371	CC	携带 CC 基因型的乳腺癌患者，与 TT 或 TC 基因型相比，在使用环磷酰胺和多柔比星治疗时，剂量延迟的可能性可能会降低。白细胞减少和中性粒细胞减少是剂量延迟的最常见原因。[PMID:20179710]	3
环磷酰胺 + 多柔比星 + 氟尿嘧啶	XRCC1	rs25487	CT	携带 CT 基因型乳腺癌患者，与 CC 基因型相比，在使用环磷酰胺、多柔比星和氟尿嘧啶(FAC)治疗时，恶心的风险可能会增加。[PMID:29507678]	3
环磷酰胺 + 多柔比星 + 氟尿嘧啶	CYP1B1	rs1056836	GG	携带 GG 基因型乳腺癌患者，与 CC 基因型相比，在使用环磷酰胺、多柔比星和氟尿嘧啶（FAC）治疗时，恶心的风险可能会降低。	3

化疗药物	检测基因	检测位点	检测结果	毒副作用风险用药提示(仅供参考)	等级
				[PMID:29507678]	
环磷酰胺 + 多柔比星 + 氟尿嘧啶	ABCB1	rs1045642	GG	携带 GG 基因型的乳腺癌患者，相比于 AA 或 AG 基因型，在接受环磷酰胺、多柔比星和氟尿嘧啶（FAC）治疗时发生贫血的风险可能会增加。[PMID:29507678]	3
环磷酰胺 + 多柔比星 + 氟尿嘧啶	GSTP1	rs1695	AG	携带 AG 基因型的乳腺癌患者，相比于 GG 基因型，在使用环磷酰胺、多柔比星和氟尿嘧啶(FAC)治疗时，恶心的风险可能会增加。[PMID:29507678]	3
环磷酰胺 + 多柔比星 + 氟尿嘧啶	ABCC4	rs9561778	GT	携带 GT 基因型的乳腺癌患者，相比于 GG 基因型，在使用 CAF(环磷酰胺、多柔比星和氟尿嘧啶)治疗时，发生不良反应的风险可能增加。[PMID:19696793]	3
环磷酰胺 + 多柔比星 + 氟尿嘧啶	ERCC1	rs3212986	CC	携带 CC 基因的乳腺癌患者，与 AA 基因型相比，在使用环磷酰胺、多柔比星和氟尿嘧啶（FAC）治疗时，中性粒细胞减少的风险可能降低。[PMID:29507678]	3
环磷酰胺 + 多柔比星 + 氟尿嘧啶	ERCC1	rs11615	GG	携带 GG 基因型的乳腺癌患者，与 AA 基因型患者相比，在使用环磷酰胺、多柔比星和氟尿嘧啶（FAC）治疗时，贫血和肾毒性的风险可能增加。[PMID:29507678]	3
吉西他滨	SLC28A1	rs2242046	GG	携带 GG 基因型的非小细胞肺癌患者，与 AA 或 AG 基因型患者相比，吉西他滨治疗时血液毒性的风险可能降低。[PMID:18538445]	3
吉西他滨	RRM1	rs1042858	AG	携带 AG 基因型结合 rs9937 位点的 G 等位基因的乳腺癌患者，相比于 GG 基因型，接受吉西他滨治疗可能会降低包括中性粒细胞减弱症在内的副作用的风险。[PMID:17602053, PMID:17602053]	4
甲氨蝶呤	MTHFR	rs1801133	AG	携带 rs1801133 AG 基因型癌症患者，与 GG 基因型患者相比，接受甲氨蝶呤治疗的毒性风险可能增加。然而，相互矛盾的证据已被报道[PMID:25110820, PMID:17488658, PMID:27781293]	2A
甲氨蝶呤	ABCB1	rs1128503	GG	携带 GG 基因型的患者，与 AA 或 AG 基因型患者相比，甲氨蝶呤治疗时发生副作用的风险可能较低。然而，报道的证据相互矛盾。[PMID:34346513, PMID:25007187, PMID:28525903]	3
甲氨蝶呤	ABCB1	rs1045642	GG	携带 GG 基因型的骨肉瘤患者，与 AA 或 AG 基因型相比，在甲氨蝶呤治疗时发生粘膜炎的风险降低相关。然而，报道的证据相互矛盾。[PMID:21887680, PMID:34346513]	3
卡铂	MTR	rs1805087	AA	携带 AA 基因型癌症患者，与 GG 基因型患者相比，可能具有较低的药物毒性风险和较低的顺铂或卡铂治疗反应。[PMID:29662106, PMID:19159907, PMID:21605004]	3
卡培他滨	DPYD	rs3918290	CC	携带 CC 基因型的癌症患者，相比于 TT 或 CT 基因型，接受卡培他滨（一种以氟嘧啶为基础的化疗）治疗可能降低药物毒性的风险和严重程度。然而，报道的证据相互矛盾。[PMID:30114658, PMID:21498394, PMID:30723313]	1A
卡培他滨	ABCB1	rs1128503	GG	携带 GG 基因型的结直肠癌患者，与 AA 基因型患者相比，接受卡培他滨治疗时，发生中性粒细胞减少或手足综合征的风险可能增加。[PMID:21142915]	3
卡培他滨	DPYD	rs67376798	TT	携带 TT 基因型的肿瘤患者，与 AA 或 AT 基因型相比，在使用卡培他滨（一种基于氟嘧啶的化疗）治疗时，可能有更低的药物毒性风险和较轻的药物毒性。然而，据报道有相互矛盾的证据。[PMID:29152729, PMID:31745289, PMID:24590654]	1A
卡培他滨	DPYD	rs55886062	AA	携带 AA 基因型癌症患者，与 AC 或 CC 基因型患者相比，接受卡培他滨（一种以氟嘧啶为基础的化疗）治疗的药物毒性风险可能降低。然而，报道的证据相互矛盾。[PMID:29152729, PMID:31745289, PMID:30485432]	1A
卡培他滨	ABCB1	rs1045642	GG	携带 GG 基因型的结直肠癌患者，相比于 AA	3

化疗药物	检测基因	检测位点	检测结果	毒副作用风险用药提示(仅供参考)	等级
				基因型，使用卡培他滨治疗手足综合征的风险可能增加。[PMID:21142915]	
卡培他滨	DPYD	rs12022243	CC	携带 CC 基因型的结直肠癌患者，与 TT 基因型相比，在使用卡培他滨治疗时发生药物毒性，特别是腹泻的风险可能降低。[PMID:24647007]	3
卡培他滨	DPYD	rs76387818	GG	携带 GG 基因型的结直肠癌患者，相比于 AA 基因型，卡培他滨治疗时发生药物毒性，特别是手足综合征的风险可能会降低。[PMID:24647007]	3
帕洛诺司琼	ERCC1	rs3212986	CC	携带 CC 基因的肿瘤患者，与 AA 基因型相比，在使用格拉司琼或帕洛诺司琼治疗时在顺铂给药后的前 24 小时内可能有更好的反应和呕吐减少。[PMID:29177570]	3
培美曲塞	DHFR	rs1650697	AG	携带 AG 基因型的非小细胞肺癌和间皮瘤患者，相比于 GG 基因型，在使用培美曲塞治疗时发生药物毒性的风险可能更高。[PMID:24732178]	3
培美曲塞	DHFR	rs442767	GT	携带 GT 基因型的非小细胞肺癌患者，与 GG 基因型相比，在使用培美曲塞治疗时出现疲劳的风险可能会降低。[PMID:23709418]	3
培美曲塞	GGH	rs11545078	GG	携带 GG 基因型的非小细胞肺癌和间皮瘤患者，与 AA 基因型相比，在使用培美曲塞治疗时发生药物毒性的风险可能更高。[PMID:24732178]	3
顺铂	TPMT	rs1142345	TT	携带 TT 基因型的儿童癌症患者，与 TC 或 CC 基因型儿童相比，接受顺铂治疗的听力损失风险可能降低。然而，多项研究发现该 SNP 与顺铂诱发的耳毒性风险之间没有关联。[PMID:23588304, PMID:25141953, PMID:19898482]	3
顺铂	GSTP1	rs1695	AG	携带 AG 基因型的各种癌症患者，相比于 AA 基因型，在使用顺铂为基础的方案治疗时，耳毒性的风险可能增加。然而，相互矛盾的证据已被报道。[PMID:17228018, PMID:28448657, PMID:23065688]	3
顺铂	TPMT	rs12201199	AA	携带 AA 基因型癌症儿童患者，相比于 TT 基因型儿童，在顺铂治疗中发生听力损失的风险可能较低。然而，多项研究发现该 SNP 与顺铂诱发的耳毒性风险之间没有关联[PMID:23588304, PMID:19898482, PMID:25141953]	3
顺铂	SLC22A2	rs316019	CC	携带 CC 基因型的肿瘤患者，与 AC 基因型患者相比，在使用顺铂治疗时的反应可能会增加肾毒性的风险（通过血清肌酐测定），但与其他生物标志物的研究显示了相互矛盾的结果。[PMID:28640195, PMID:19625999, PMID:21902499]	3
顺铂	XPC	rs2228001	GT	携带 GT 基因型的骨肉瘤患者，与 TT 基因型患者相比，在顺铂治疗中发生耳毒性的风险可能更高。[PMID:19434073]	3
顺铂	ERCC1	rs11615	GG	携带 GG 基因型的肿瘤患者，与 AA 或 AG 基因型相比，在使用顺铂治疗时可能与肾毒性降低有关。[PMID:21902499]	3
顺铂	XPC	rs2228001	GT	携带 GT 基因型的尿道膀胱癌症患者，与 TT 基因型相比，在顺铂治疗时发生中性粒细胞减少和产生药物毒性的风险可能更高。[PMID:21047201]	3
顺铂	ERCC1	rs3212986	CC	携带 CC 基因的肿瘤患者，与 AA 或 AC 基因型相比，在使用顺铂治疗时可能增加肾毒性风险。[PMID:21902499, PMID:19786980, PMID:22188361]	3
顺铂	TPMT	rs1800460	CC	携带 CC 基因型的儿童癌症患者，相比于 CT 或 TT 基因型，接受顺铂治疗的听力损失风险可能降低失。然而，多项研究发现该 SNP 与顺铂诱发的耳毒性风险之间没有关联。[PMID:23588304, PMID:25141953, PMID:19898482]	3
顺铂	MTR	rs1805087	AA	携带 AA 基因型癌症患者，与 GG 基因型患者相比，可能具有较低的药物毒性风险和较低的顺铂或卡铂治疗反应。[PMID:29662106, PMID:19159907, PMID:21605004]	3

化疗药物	检测基因	检测位点	检测结果	毒副作用风险用药提示(仅供参考)	等级
顺铂 + 多柔比星	ERCC1	rs11615	GG	携带 GG 基因型的骨肉瘤患者，与 AA 基因型相比，在使用顺铂和多柔比星治疗时发生粘膜炎的风险可能增加。[PMID:21887680]	3
顺铂 + 多柔比星 + 甲氨蝶呤	ERCC2	rs13181	TT	携带 TT 基因的骨肉瘤患者，与 GG 或 GT 基因型相比，在使用顺铂、多柔比星和甲氨蝶呤治疗时发生肾毒性的风险可能降低。[PMID:21887680]	3
顺铂 + 多柔比星 + 甲氨蝶呤	GSTP1	rs1695	AG	携带 AG 基因型的骨肉瘤患者，相比于 AA 基因型，对顺铂、多柔比星和甲氨蝶呤治疗的反应可能降低，发生心脏毒性的风险可能更高。[PMID:21887680]	3
顺铂 + 多柔比星 + 甲氨蝶呤	MTHFR	rs1801133	AG	携带 AG 基因型骨肉瘤患者，与 GG 基因型相比，在使用顺铂、多柔比星和甲氨蝶呤呤治疗时发生肾毒性的风险可能增加。[PMID:21887680]	3
顺铂 + 环磷酰胺	ERCC1	rs11615	GG	携带 GG 基因型的卵巢癌患者，与 AG 基因型患者相比，在接受顺铂和环磷酰胺治疗时的肾毒性风险可能降低。这一关联在一些研究中遭到了反驳。[PMID:19786980, PMID:22188361]	3
顺铂 + 伊立替康	UGT1A1	rs4148323	AG	携带 AG 基因型的非小细胞肺癌患者，与 AA 基因型的患者相比，接受顺铂和伊立替康治疗时，发生中性粒细胞减少的风险可能降低。[PMID:16636344]	1B
顺铂 + 伊立替康	UGT1A1	rs4148323	AG	携带 AG 基因型的癌症患者，与 GG 基因型的患者相比，接受顺铂和伊立替康治疗时，发生腹泻和中性粒细胞减少的风险可能增加。[PMID:19299905]	1B
叶酸 + 替吉奥	UMPS	rs1801019	GG	携带 GG 基因型的结直肠癌患者,相比于 CC 基因型，在使用叶酸和替吉奥治疗时，毒性风险可能降低。[PMID:28347333, PMID:20647221]	3
伊立替康	C8orf34	rs1517114	GG	携带 GG 基因的非小细胞肺癌患者，与 CC 或 CG 基因型相比，在使用伊立替康治疗时，腹泻严重程度可能会降低。[PMID:22664479]	3
伊立替康	UGT1A1	rs4148323	AG	携带 AG 基因型的癌症患者，与 GG 基因型的患者相比，接受伊立替康治疗时，发生腹泻和中性粒细胞减少的风险可能增加。然而，相互矛盾的证据已被报道。[PMID:28502040, PMID:19299905, PMID:28367249]	1B
伊立替康	SEMA3C	rs7779029	TT	携带 TT 基因型的非小细胞肺癌患者，相比于 CC 或 CT 基因型，在接受伊立替康治疗时中性粒细胞减少的严重程度可能降低。[PMID:22664479]	3
紫杉醇	SOD2	rs4880	AA	携带 AA 基因型乳腺癌患者,与 AG 或 GG 基因型患者相比，在紫杉醇治疗时发生神经病变的风险可能会增加。[PMID:25495407]	3
紫杉醇	ERCC1	rs3212986	CC	携带 CC 基因型的乳腺癌患者，相比于 AA 或 AC 基因型，在使用紫杉醇治疗时神经病变的风险可能降低。[PMID:25495407]	3
紫杉醇	ABCB1	rs1128503	GG	携带 GG 基因型乳腺癌患者，与 AA 基因型相比，使用紫杉醇治疗，周围神经病变的严重程度可能降低。[PMID:34280009, PMID:28447211]	3
紫杉醇	CYP2C8	rs1934951	CC	携带 CC 基因型的乳腺癌患者，相比于 CT 或 TT 基因型，使用紫杉醇治疗可能会降低贫血的风险。[PMID:25495407]	4
紫杉烷 + 铂类	ABCB1	rs2032582	TT	携带 TT 基因型的卵巢癌患者，相比于 CC 基因型，紫杉烷和铂类方案的胃肠道毒性风险可能更高。[PMID:19203783]	3

化疗药物有效性解析

化疗药物	检测基因	检测位点	检测结果	有效性用药提示(仅供参考)	等级
阿那曲唑	CYP19A1	rs727479	AC	携带 AC 基因型的乳腺癌患者,相比于 AA 基因型，在接受阿那曲唑治疗时，乳腺癌复发的可能性可能会增加(无复发生存期降低)。[PMID:27747906]	3
奥沙利铂	ABCB1	rs1045642	GG	携带 GG 基因型的结直肠癌患者，相比于 AA 基因型，在接受奥沙利铂为基础的化疗治疗时，无复发生存期无统计学差异。[PMID:23746184]	3
奥沙利铂	TP53	rs1042522	CC	携带 CC 基因型的结直肠癌患者,相比于 CG 和 GG 基因型，对奥沙利铂的应答增加相关。[PMID:29925895]	3
奥沙利铂	ABCB1	rs1128503	GG	携带 GG 基因型的结直肠癌患者，与 AA 或 AG 基因型相比，在接受以奥沙利铂为基础的化疗治疗时，总生存期可能会缩短。[PMID:23746184]	3
表柔比星 ＋ 氟尿嘧啶 ＋ 奥沙利铂	NQO1	rs1800566	GG	携带 GG 基因型的转移性胃癌患者，相比于 AA 或 AG 基因型，对表柔比星、氟尿嘧啶和奥沙利铂治疗的反应可能更高。[PMID:25545243]	3
铂类	ERCC2	rs13181	TT	携带 TT 基因型的结直肠癌患者，与 GG 基因型相比，在使用铂类治疗后的死亡风险降低。[PMID:15213713]	3
铂类	ABCB1	rs1045642	GG	携带 GG 基因型的非小细胞肺癌患者，相比于 AA 或 AG 基因型，可能对铂基化疗有更好的反应。[PMID:22761669]	3
铂类	ERCC1	rs3212986	CC	携带 CC 基因型的卵巢癌患者，相比于 AC 或 AA 基因型，在使用铂类治疗时的生存率可能会增加。[PMID:19203783]	3
铂类	GSTP1	rs1695	AG	携带 AG 基因型的癌症患者，相比于 AA 基因型，在接受铂类药物治疗时可能具有更低的毒性风险。[PMID:15213713, PMID:17409936, PMID:27995989]	3
铂类	GSTP1	rs1695	AG	-	-
铂类	XRCC1	rs25487	CT	携带 rs25487 CT 基因型的癌症患者，与 CC 基因型相比，在接受铂类化疗治疗时，其药物反应可能会降低。然而，报道的证据相互矛盾。[PMID:19362955, PMID:29662106, PMID:24446315]	2B
蒽环类 ＋ 紫杉烷	ABCB1	rs1045642	GG	携带 GG 基因型的乳腺癌患者，相比于 AA 基因型，在使用蒽环类药物及相关物质和紫杉类药物治疗时，完全缓解的可能性可能降低。[PMID:12684679]	3
氟尿嘧啶 ＋ 奥沙利铂	ERCC2	rs13181	TT	携带 TT 基因型的结直肠癌患者，与 GT 基因型相比，在使用氟尿嘧啶和奥沙利铂治疗时的无进展生存期降低相关。[PMID:21449681]	3
氟尿嘧啶 ＋ 亚叶酸	ERCC2	rs13181	TT	携带 TT 基因型的结直肠癌患者，与 GG 基因型相比，在使用氟尿嘧啶和亚叶酸治疗时，早期复发风险可能降低。[PMID:18267032]	3
氟尿嘧啶 ＋ 亚叶酸 ＋ 奥沙利铂	MTHFR	rs1801133	AG	携带 AG 基因型结直肠癌患者，与 GG 基因型相比，在使用氟尿嘧啶、亚叶酸和奥沙利铂（FOLFOX 疗法）治疗时的反应可能增加。然而，存在相互矛盾的证据。[PMID:29134491, PMID:20078613, PMID:24980946]	3
氟尿嘧啶 ＋ 亚叶酸 ＋ 奥沙利铂	ERCC2	rs13181	TT	携带 TT 基因型的结直肠癌患者，与 GG 基因型相比，在使用氟尿嘧啶、亚叶酸和奥沙利铂治疗时，可能 1）药物毒性风险降低；2）无进展生存期增加。[PMID:20078613, PMID:20385995]	3
氟尿嘧啶 ＋ 亚叶酸 ＋ 奥沙利铂	ERCC1	rs11615	GG	携带 GG 基因型的结直肠癌患者，与 AA 或 AG 基因型相比，在使用 FOLFOX（氟尿嘧啶、亚叶酸、奥沙利铂）治疗时，总生存期和无进展生存期可能会增加。[PMID:21057378]	3
格拉司琼	ERCC1	rs3212986	CC	携带 CC 基因的肿瘤患者，与 AA 基因型相比，在使用格拉司琼或帕洛诺司琼治疗时在顺铂给药后的前 24 小时内可能有更好的反应和呕吐减少。[PMID:29177570]	3
格拉司琼	ABCB1	rs1045642	GG	携带 GG 基因型的癌症患者，相比于 AA 基因型的患者相比，在使用格拉司琼治疗时避免化疗引起的恶心呕吐(CINV)的可能性更小，尽管这在一项研究中是矛盾的。[PMID:27241063,	3

化疗药物	检测基因	检测位点	检测结果	有效性用药提示(仅供参考)	等级
				PMID:29177570]	
环磷酰胺	SOD2	rs4880	AA	携带 AA 基因型乳腺癌患者，与 GG 基因型相比，接受环磷酰胺治疗可能会增加生存率。[PMID:19509150]	3
环磷酰胺	GSTP1	rs1695	AG	携带 AG 基因型的肿瘤患者，相比于 GG 基因型，对环磷酰胺的反应可能增加。然而，相互矛盾的证据已被报道。[PMID:19786980, PMID:21362365, PMID:20568049]	3
环磷酰胺 ＋ 表柔比星	GSTP1	rs1695	AG	携带 AG 基因型的乳腺癌患者，相比于 GG 基因型，在使用环磷酰胺和表柔比星治疗时，毒性的严重程度可能会降低，反应可能更高。[PMID:21362365]	3
环磷酰胺 ＋ 表柔比星 ＋ 氟尿嘧啶	CYP1B1	rs1056836	GG	携带GG基因型乳腺癌患者,相比于CC基因型,在使用环磷酰胺、表柔比星和氟尿嘧啶治疗时可能有更好的疗效。[PMID:24958282]	3
环磷酰胺 ＋ 表柔比星 ＋ 氟尿嘧啶	NQO1	rs1800566	GG	携带 GG 基因型的乳腺癌患者，相比于 AA 基因型，对环磷酰胺、表柔比星和氟尿嘧啶治疗的反应可能更高。[PMID:18511948]	3
环磷酰胺 ＋ 多柔比星	ABCB1	rs2032582	TT	携带 TT 基因型的乳腺癌患者，相比于 CC 基因型，在使用环磷酰胺和多柔比星治疗时可能会降低生存率。[PMID:20179710, PMID:29938344]	3
卡铂	MTR	rs1805087	AA	携带 AA 基因型癌症患者，与 GG 基因型患者相比，可能具有较低的药物毒性风险和较低的顺铂或卡铂治疗反应。[PMID:29662106, PMID:19159907, PMID:21605004]	3
卡培他滨 ＋ 表柔比星 ＋ 铂类	GSTP1	rs1695	AG	携带 AG 基因型的结直肠癌患者，相比于 GG 基因型，对卡培他滨、表柔比星和铂类治疗的反应可能较低。[PMID:25677447]	3
卡培他滨 ＋ 顺铂 ＋ 多西他赛 ＋ 表柔比星 ＋ 吉西他滨	ERCC1	rs11615	GG	携带 GG 基因型胰腺癌患者，与 AA 基因型相比，在使用卡培他滨、顺铂、多西他赛、表柔比星和吉西他滨治疗时的反应可能降低。[PMID:22026922]	3
卡培他滨 ＋ 顺铂 ＋ 多西他赛 ＋ 表柔比星 ＋ 吉西他滨	ERCC2	rs13181	TT	携带 TT 基因型的胰腺癌患者，与 GG 基因型相比，在使用卡培他滨、顺铂、多西他赛、表柔比星和吉西他滨治疗后的无进展生存期和总生存期可能增加。[PMID:22026922]	3
帕洛诺司琼	ERCC1	rs3212986	CC	携带 CC 基因的肿瘤患者，与 AA 基因型相比，在使用格拉司琼或帕洛诺司琼治疗时在顺铂给药后的前 24 小时内可能有更好的反应和呕吐减少。[PMID:29177570]	3
培美曲塞	MTHFR	rs1801133	AG	携带 AG 基因型非小细胞肺癌和间皮瘤患者，与 GG 基因型相比，在使用培美曲塞治疗时的总生存率降低。[PMID:24732178]	3
培美曲塞	SLC19A1	rs1051298	GG	携带 GG 基因型的非小细胞肺癌和间皮瘤患者，与 AA 或 AG 基因型的患者相比，在使用培美曲塞治疗时，其总体和无进展生存期可能更长。[PMID:24732178, PMID:19841321]	3
顺铂	GSTP1	rs1138272	CC	携带 CC 基因型的癌症患者，与 CT 或 TT 基因型患者相比，顺铂治疗可能有更长的生存期。然而，这种关联存在着相互矛盾的证据。[PMID:25592234, PMID:22188361, PMID:17409936]	3
顺铂	ERCC1	rs11615	GG	携带 GG 基因型的卵巢癌患者，与 AA 或 AG 基因型的患者相比，对顺铂的反应增加。[PMID:32173978]	3
顺铂	ERCC1	rs11615	GG	携带 GG 基因型的食管癌患者，与 AA 基因型患者相比，使用顺铂治疗后的总生存期和无进展生存期增加。[PMID:19620936]	3
顺铂	ERCC2	rs13181	TT	携带 TT 基因型的骨肉瘤患者，与 GG 或 GT 基因型相比，在使用顺铂治疗后无事件生存率增加。[PMID:19434073]	3
顺铂	ERCC2	rs13181	TT	携带 TT 基因型的食管癌患者，与 GG 基因型相比，在使用顺铂治疗后与总生存期和无进展生存期减少。[PMID:19620936]	3
顺铂	MTR	rs1805087	AA	携带 AA 基因型癌症患者，与 GG 基因型患者相比，可能具有较低的药物毒性风险和较低的顺铂或卡铂治疗反应。[PMID:29662106, PMID:19159907, PMID:21605004]	3
顺铂 ＋ 多柔比星 ＋ 甲	GSTP1	rs1695	AG	携带 AG 基因型的骨肉瘤患者，相比于 AA 基	3

化疗药物	检测基因	检测位点	检测结果	有效性用药提示(仅供参考)	等级
氨蝶呤				因型，对顺铂、多柔比星和甲氨蝶呤治疗的反应可能降低，发生心脏毒性的风险可能更高。[PMID:21887680]	
顺铂 + 多柔比星 + 异环磷酰胺 + 甲氨蝶呤	ERCC2	rs13181	TT	携带 TT 基因型的骨肉瘤患者，与 GG 基因型相比，在使用顺铂、多柔比星、异环磷酰胺和甲氨蝶呤治疗时无事件生存率降低。[PMID:21826087]	3
顺铂 + 环磷酰胺	GSTP1	rs1695	AG	携带 AG 基因型的卵巢肿瘤患者，相比于 AA 基因型，接受顺铂和环磷酰胺治疗的无进展生存的可能性可能降低。然而，这种关联在其他研究中被反驳了。[PMID:19786980, PMID:22188361]	3
顺铂 + 环磷酰胺	TPMT	rs1142345	TT	携带 TT 基因型的卵巢癌患者，与 CT 基因型患者相比，接受顺铂和环磷酰胺的化疗可能增加无进展生存期。[PMID:24533712]	3
顺铂 + 环磷酰胺 + 多柔比星 + 甲氨蝶呤 + 长春新碱	ABCB1	rs1128503	GG	携带 GG 基因型的骨肉瘤患者，与 AA 或 AG 基因型患者相比，在接受顺铂、环磷酰胺、多柔比星、甲氨蝶呤和长春新碱治疗时，死亡风险可能增加。[PMID:22016816]	3
顺铂 + 吉西他滨	ERCC2	rs13181	TT	携带 TT 基因型的间皮瘤患者，与 GG 和 GT 基因型相比，在使用顺铂和吉西他滨治疗时的总体生存率和无进展生存率可能有所提高。[PMID:28422153]	3
顺铂 + 吉西他滨	ERCC1	rs3212986	CC	携带 CC 基因型的间皮瘤患者，与 AA 和 AC 基因型相比，在使用顺铂和吉西他滨治疗时的总体生存率和无进展生存率可能有所提高。[PMID:28422153]	3
顺铂 + 吉西他滨	ERCC1	rs11615	GG	携带 GG 基因型的非小细胞肺癌患者，与 AA 基因型相比，在使用顺铂和吉西他滨治疗时的反应可能降低。[PMID:18347182]	3
顺铂 + 依托泊苷	NQO1	rs1800566	GG	携带 GG 基因型的非小细胞肺癌患者，相比于 AA 基因型，使用顺铂和依托泊苷治疗时总生存率增加。[PMID:21479364]	3
顺铂 + 紫杉醇	TP53	rs1042522	CC	携带 CC 基因型的胃癌患者，相比于 CG 和 GG 基因型，在接受顺铂和紫杉醇治疗时，其一年无进展生存期增加。[PMID:19052714]	3
他莫昔芬	CYP19A1	rs4646	AC	携带 AC 基因型乳腺癌女性患者，与 CC 基因型的患者相比，在接受他莫西芬治疗时，无病生存期可能增加。[PMID:25793413, PMID:26191232]	3
他莫昔芬	CYP19A1	rs4646	AC	携带 AC 基因型的乳腺癌绝经后妇女，与 AA 基因型的患者相比，在接受他莫西芬治疗时，无病生存期可能增加。[PMID:25793413, PMID:26191232]	3
他莫昔芬	ABCB1	rs1045642	GG	携带 GG 基因型的乳腺癌患者，相比于 AG 基因型，在接受他莫昔芬治疗时，其疾病复发的几率可能会降低。[PMID:24019753]	3
伊立替康	SLCO1B1	rs2306283	GG	携带 GG 基因型的结直肠癌患者，相比于 AA 和 AG 基因型，可能会对伊立替康有较慢的响应，较短的 PFS，伊立替康相关的治疗维持时间较短。[PMID:24143213]	3
紫杉醇	ABCB1	rs2032582	TT	携带 TT 基因型的乳腺癌和卵巢癌患者，相比于其他基因型，对紫杉醇的反应可能增加。[PMID:18836089, PMID:16467099]	3

证据等级说明：

1A：相关性已被临床药理学联合会或某些重大卫生系统认可。

1B：相关性基于多项有统计显著性的研究，此相关性必须在多于一个群体的研究中被重复，且显著性较强。

2A：PharmGKB 定义的 2B 级之上的有 VIP（Very Important Pharmacogene，非常重要的用药相关基因）的变异。2A 级变异为已知用药相关基因，与 2B 级相比功能相关性更显著。

2B：相关性基于多项重复性研究所得的中等程度证据，但其中某些研究可能缺乏统计学显著性或规模效应较小。

3 ：相关性基于单一显著性（尚无重复性）研究或多项缺乏明确证据的研究。

上述结果所代表的含义如有疑问请咨询临床医生。

检测人：袁天怡　　　　报告审核：沈佳丹

启东领星医学检验实验室有限公司 报告专用章 3206812070668

报告时间：2023-05-15

---

1. 本实验室检测使用Illumina 高通量测序平台技术对样本进行杂交捕获测序分析。
2. 适用性说明：

   (a) 本检测只适用于指定肿瘤基因的DNA水平的变异（包括单核苷酸变异、短片段插入或缺失，拷贝数变异和断点发生在产品捕获范围内的基因重排），不涉及DNA甲基化、蛋白质水平分析、RNA水平分析；

   (b) 肿瘤是一种复杂的系统性疾病，评定其是否为恶性需要由临床医生经过多方检验结果进行判定。因此本检测不能用于确认或排除恶性肿瘤的存在；

   (c) 检测结果只在检测范围内针对本次送检样本负责，检测结果仅供医生科研参考，不构成临床诊疗的全部或唯一依据。就临床诊疗方案或其他疑问，请咨询执业医师意见并以之为准。
3. 本报告涉及的基因序列版本号为Grch37/hg19。
4. 局限性说明：

   受限于以下因素，本次检测结果存在一定的局限性。请仔细阅读以下局限性说明，如有任何疑问，请咨询执业医师意见并以之为准：

   (a) 科学数据表明，部分患者不存在明确的靶向药物对应的基因突变，所以并非所有受检者都可以找到对应靶向药物。

   (b) 在肿瘤发展或治疗过程中可能出现的获得性基因组变异会使癌症的突变谱发生变化，同时肿瘤也可能存在肿瘤间和肿瘤内的异质性。利用ctDNA或小块组织检测到的变异可能无法完全反映病灶部位全部细胞变异情况。阴性结果并不能排除样本存在检测范围之外的其他基因突变。检测到靶向药物相关变异也不意味着患者一定能从靶向治疗中获益；

   (c) 因患者个体差异或其他一些不可控原因，可能出现假阳性或假阴性的检测结果，需结合其他检查方法综合分析，以更准确、全面地反映受检者的实际情况；

   (d) 因当前生物技术及检测水平的限制以及肿瘤细胞的复杂性，本次检测结果并非完全准确，必要时请及时进行二次检测确认；

   (e) 检测结果仅基于当前的权威数据库和专业文献做出，随着生物技术的发展，检测结果可能因相关参考依据更新而发生改变，该等改变将不包含在本次检测的参考范围内。
5. 依据相关法律、法规的要求，受检者的个人信息及检测结果将予以严格保密，未经受检者本人同意不会向第三方提供（未免疑义，为开展检测服务所必要的、向其关联方提供和披露的除外）。如因科学研究或其他需要而需使用或向第三方提供受检者的个人信息或检测数据时，我们将依照相关法律、法规的要求，另行征得受检者同意，或对其个人信息进行匿名化处理，以确保不会识别到受检者本人。

附录

样本质控情况

质控环节	质控参数	质控数值	质控标准
病理评估	肿瘤细胞含量	50%	≥10%
核酸质量评估	DNA总量（ng）	1182	≥100
	DNA降解程度	C	A/B/C级
	预文库总量（ng）	2763	≥500
测序质量评估	平均测序深度	1474	≥500
	覆盖均一性	99%	≥90%
	基因组比对率	99%	≥95%
	碱基质量Q30 占比	88%	≥80%
总体质量评估	合格		

1. 肿瘤细胞含量：送检样本经 HE 染色评估的肿瘤细胞含量。如送检样本不满足病理评估所需条件，则不进行此项评估。血浆 cfDNA 样本不做此项评估。

2. DNA 总量：送检样本的 DNA 提取总量。

3. DNA 降解程度：cfDNA 样本不适用于此项评估。
- A 级：有主带，或降解均匀且降解区域主要集中在 5000bp 以上；
- B 级：无主带，降解均匀且降解区域主要集中在 1500-5000bp 之间；
- C 级：无主带，降解均匀且降解区域主要集中在 500-1500bp 之间；
- D 级（不合格）：无主带，降解均匀且降解区域主要集中在 500-1500bp 之间，但总量介于 100ng 到 300ng 之间；或降解区域弥散在 150-1000bp，或集中在 500bp 以下。

4. 预文库总量：核酸片段经过接头连接和扩增纯化后中间产物的总量。

5. 平均测序深度：目标区域每个碱基被测到的平均次数。

6. 覆盖均一性：测序深度达到平均测序深度 20%的碱基占比。

7. 基因组比对率：比对到参考基因组上的 reads 数目占比。

8. 碱基质量 Q30 占比：测序数据中碱基质量在 Q30 以上（即碱基识别错误率在千分之一以下）的占比。

9. 总体质量评估：根据 DNA 平均测序深度进行评估，分为“合格”、“警戒“、”不合格”三个等级。总体质量评估为“警戒“或者“不合格”时，均会影响检测的准确性和敏感性。

常见靶向药物相关基因检测列表

基因	检测内容	靶向药物	用药提示（仅供参考）
ALK	突变融合	ALK 抑制剂：**克唑替尼*（Crizotinib）**（FDA/NMPA 批准用于非小细胞肺癌；FDA 批准用于间变性大细胞淋巴瘤）、**塞瑞替尼*（Ceritinib）**（FDA/NMPA 批准用于非小细胞肺癌）、**阿来替尼*（Alectinib）**（FDA/NMPA 批准用于非小细胞肺癌）、**布吉他滨（Brigatinib）**（FDA 批准用于非小细胞肺癌）、**劳拉替尼（Lorlatinib）**（FDA 批准用于非小细胞肺癌）、**恩沙替尼*（Ensartinib）**（NMPA 批准用于非小细胞肺癌）；<br>EGFR 抑制剂：**厄洛替尼*（Erlotinib）**（FDA/NMPA 批准用于非小细胞肺癌）、**吉非替尼*（Gefitinib）**（FDA/NMPA 批准用于非小细胞肺癌）、**阿法替尼*（Afatinib）**（FDA/NMPA 批准用于非小细胞肺癌）、**达可替尼*（Dacomitinib）**（FDA/NMPA 批准用于非小细胞肺癌）、**奥希替尼*（Osimertinib）**（FDA/NMPA 批准用于非小细胞肺癌）、**埃克替尼*（Icotinib）**（NMPA 批准用于非小细胞肺癌）	在非小细胞肺癌中，ALK 融合对 ALK TKIs 药物克唑替尼[PMID: 25470694]、塞瑞替尼[PMID: 28126333]、阿来替尼[PMID: 28586279]、布吉他滨[PMID: 30280657]、劳拉替尼[PMID: 33207094]、恩沙替尼敏感[PMID: 29563138]；对 EGFR TKIs 药物埃克替尼、厄洛替尼、吉非替尼、阿法替尼、奥希替尼、达可替尼产生耐药性[PMID: 19667264]；ALK 酪氨酸激酶结构域关键位点（包含 1151Tins、L1152R、C1156Y、F1174L、L1196M、G1202R、S1206Y、G1269A、F1174V、F1245C）发生突变会导致对一代 ALK 抑制剂克唑替尼产生耐药性[PMID: 22277784]，L1196M、G1269A、I1171T、S1206Y 位点突变对二代 ALK 抑制剂塞瑞替尼敏感[PMID: 24675041]，L1196M、G1269A、C1156Y、F1174L、1151Tins、L1152R 位点突变对二代 ALK 抑制剂阿来替尼敏感[PMID: 26579422]。炎症性肌纤维母细胞瘤中，ALK 融合对克唑替尼[PMID: 20979472]、塞瑞替尼[PMID: 24670165]、布吉他滨[PMID: 27836716]敏感；间变性大细胞淋巴瘤中，ALK 融合对克唑替尼敏感[PMID: 30140094]。

基因	检测内容	靶向药物	用药提示（仅供参考）
BRAF	突变	BRAF 抑制剂：**维莫非尼*（Vemurafenib）**（FDA/NMPA 批准用于黑色素瘤；FDA 批准用于埃德海姆-切斯特病）、**达拉非尼（Dabrafenib）**（FDA 批准用于黑色素瘤）；<br>MEK 抑制剂：**曲美替尼（Trametinib）**（FDA 批准用于黑色素瘤）；<br>**曲美替尼+达拉非尼*（Dabrafenib+Trametinib）**（FDA 批准用于非小细胞肺癌、甲状腺癌；FDA/NMPA 批准用于黑色素瘤）；<br>**康奈非尼+贝美替尼（Encorafenib+Binimetinib）**（FDA 批准用于黑色素瘤）；<br>**考比替尼+维莫非尼（Cobimetinib+Vemurafenib）**（FDA 批准用于黑色素瘤）；<br>**司美替尼（**NCCN 指南推荐用于胶质瘤）；<br>**阿替利珠单抗+考比替尼+维莫非尼（Atezolizumab+Cobimetinib+Vemurafenib）**（FDA 批准用于黑色素瘤）；<br>康奈非尼+帕尼单抗（Encorafenib+Panitumumab）（NCCN 指南推荐用于结直肠癌）；<br>**康奈非尼+西妥昔单抗（Encorafenib +Cetuximab）**（FDA 批准用于结直肠癌）；<br>康奈非尼+贝美替尼+西妥昔单抗（Encorafenib+Binimetinib+Cetuximab）（临床 III 期）；<br>维莫非尼+伊立替康+西妥昔单抗（Vemurafenib+Irinotecan+Cetuximab）（临床 II 期）；<br>维莫非尼+西妥昔单抗（Vemurafenib+Cetuximab）（临床 II 期）；<br>EGFR 单抗：**帕尼单抗（Panitumumab）**（FDA 批准用于结直肠癌）、**西妥昔单抗*（Cetuximab）**（FDA/NMPA 批准用于结直肠癌；FDA 批准用于头颈部鳞状细胞癌）	肺癌中，BRAF V600E 突变对 BRAF 抑制剂维莫非尼、达拉非尼敏感[PMID: 29320312; PMID: 27080216]；黑色素瘤中，BRAF V600 突变对 BRAF 抑制剂维莫非尼、达拉非尼敏感，携带 BRAF V600 突变的患者对贝美替尼敏感，BRAF V600E/K 突变的患者对康奈非尼+贝美替尼[J Clin Oncol. 2013; 31(suppl 15): abstr 9029); PMID: 29573941]、考比替尼+维莫非尼 [PMID: 25265494; PMID: 27480103]、曲美替尼+达拉非尼[PMID: 26811525]、阿替利珠单抗+考比替尼+维莫非尼敏感[PMID: 32534646]；在结直肠癌中，BRAF V600E 突变的患者对康奈非尼+帕尼单抗、康奈非尼+西妥昔单抗、康奈非尼+贝美替尼+西妥昔单抗、维莫非尼+伊立替康+西妥昔单抗敏感[PMID: 30892987; PMID: 31566309; J Clin Oncol. 2020; 38(suppl 4): abstr 8; PMID: 33356422]，携带 BRAF V600 的患者对维莫非尼+西妥昔单抗敏感[PMID: 26287849]，携带 BRAF V600 的患者使用帕尼单抗、西妥昔单抗产生耐药性[PMID: 25673558]；对于 BRAF V600 激活突变（包括 V600E、V600K、V600R、V600D 等）的转移性黑色素瘤患者，BRAF/MEK 抑制剂组合（达拉非尼+曲美替尼或维莫非尼+考比替尼）优于 BRAF 抑制剂单药治疗[PMID: 28475671; PMID: 27480103]；在非小细胞肺癌中，达拉非尼联合曲美替尼可作为具有 BRAF V600E 突变的非小细胞肺癌患者的一线治疗方案，联合治疗不耐受的患者建议使用单药达拉非尼或维莫非尼进行治疗[PMID: 28919011]；BRAF V600E 或 V600K 突变的不可手术切除的转移黑色素瘤对曲美替尼联合达拉非尼敏感[PMID: 26811525]；BRAF V600E 或 V600K 突变的黑色素瘤对考比替尼联合维莫非尼敏感[PMID: 25265494; PMID: 27480103]。在胶质瘤中，携带 BRAF V600 激活突变和 BRAF 融合突变的患者对司美替尼敏感[PMID: 31151904]。

基因	检测内容	靶向药物	用药提示（仅供参考）
EGFR	突变	EGFR 抑制剂：**厄洛替尼*（Erlotinib）**（FDA/NMPA 批准用于非小细胞肺癌）、**吉非替尼*（Gefitinib）**（FDA/NMPA 批准用于非小细胞肺癌）、**阿法替尼*（Afatinib）**（FDA/NMPA 批准用于非小细胞肺癌）、**达可替尼*（Dacomitinib）**（FDA/NMPA 批准用于非小细胞肺癌）、**奥希替尼*（Osimertinib）**(FDA/NMPA 批准用于非小细胞肺癌)、**埃克替尼*(Icotinib)**(NMPA 批准用于非小细胞肺癌）、**阿美替尼*（Almonertinib）**（NMPA 批准用于非小细胞肺癌）；**伏美替尼*（Furmonertinib）**(NMPA 批准用于非小细胞肺癌);<br>**Amivantamab**（FDA 批准用于非小细胞肺癌）；<br>**Mobocertinib**（FDA 批准用于非小细胞肺癌）；<br>**奈西单抗+吉西他滨+顺铂（Necitumumab+Gemcitabine+Cisplatin）**（FDA 批准用于肺鳞癌）；<br>**厄洛替尼+雷莫西尤单抗（Erlotinib+Ramucirumab）**（FDA 批准用于非小细胞肺癌）；<br>厄洛替尼+贝伐珠单抗(Erlotinib+Bevacizumab)(NCCN 推荐用于非鳞非小细胞肺癌、肾癌）；<br>凡德他尼+多西他赛（Vandetanib+Docetaxel）（临床 III 期）；吉非替尼 + 阿帕替尼 （临床 III 期）；安罗替尼（临床 III 期）；Poziotinib（临床 II 期）；<br>EGFR 单抗：**西妥昔单抗*（Cetuximab）**（FDA/NMPA 批准用于结直肠癌；FDA 批准用于头颈部鳞状细胞癌）、**帕尼单抗（Panitumumab）**（FDA 批准用于结直肠癌）	在非小细胞肺癌中，EGFR 敏感突变（L858R、19 外显子插入或缺失、L861Q、G719X、S768I 等）对 EGFR TKIs（吉非替尼、厄洛替尼、埃克替尼、达可替尼）、厄洛替尼+雷莫西尤单抗[PMID: 31591063]、吉非替尼+阿帕替尼[PMID: 34033974]敏感，EGFR T790M 对 EGFR TKIs 产生耐药性，但 EGFR T790M 对 EGFR TKIs（奥希替尼[PMID: 29151359]、阿美替尼[PMID: 32916310]、伏美替尼[J Clin Oncol. 2020; 38(suppl 15): abstr 9602]）敏感，携带 EGFR 20 号外显子插入突变的患者对 Poziotinib [PMID: 29686424; PMID: 29162564]、Amivantamab [PMID: 32414908]、Mobocertinib[PMID: 34647988]敏感，EGFR 扩增的患者对凡德他尼+多西他赛[PMID: 25057173]敏感；在非鳞非小细胞肺癌中，EGFR 敏感突变对厄洛替尼+贝伐珠单抗敏感[PMID: 30975627]；在结直肠癌中，EGFR 扩增的患者对帕尼单抗敏感[PMID: 17664472]；在胶质瘤中，EGFR 扩增的患者对西妥昔单抗敏感[PMID: 22752145]；在肺鳞癌中，EGFR 扩增的患者对奈西单抗+吉西他滨+顺铂敏感[PMID: 29158193];在肺腺癌中,EGFR T790M 对安罗替尼敏感[PMID: 30578392]。
FGFR1	扩增	Infigratinib（临床 I 期）；<br>Derazantinib（临床 I 期）；<br>多韦替尼（Dovitinib）（临床 I 期）	在肾上腺皮质癌中，携带 FGFR1 扩增的患者对 Derazantinib 敏感[J Clin Oncol. 2015; 33(suppl 15): abstr 2545]；在肺鳞癌中，携带 FGFR1 扩增的患者对 Infigratinib 敏感[PMID: 27870574]；在 HER2 阳性乳腺癌中，携带 FGFR1 扩增的患者对多韦替尼敏感[PMID: 23658459]。
FGFR2	融合<br>突变<br>扩增	**Pemigatinib**（FDA 批准用于胆管癌）；<br>**Erdafitinib**（FDA 批准用于膀胱尿路上皮癌）；<br>AZD4547（临床 II 期）；<br>**Infigratinib**（FDA 批准用于胆管癌）	在胆管癌中,FGFR2 融合的患者对 Pemigatinib [PMID: 32203698]、Infigratinib 敏感[PMID: 29182496]；在胃食管结合腺癌中，FGFR2 扩增对 AZD4547 敏感[PMID: 27179038];在尿路上皮癌中，FGFR2 基因融合/突变的患者对 Erdafitinib 敏感[PMID: 31340094]。

基因	检测内容	靶向药物	用药提示（仅供参考）
**ERBB2 (HER2)**	突变<br>扩增	**奈拉替尼*（Neratinib）**（FDA/NMPA 批准用于乳腺癌）、拉帕替尼（Lapatinib）（NCCN 指南推荐用于乳腺癌）；曲妥珠单抗+帕妥珠单抗+透明质酸酶（Trastuzumab+Pertuzumab+Hyaluronidase）（NCCN 指南推荐用于乳腺癌）；<br>EGFR 抑制剂：**埃克替尼*（Icotinib）**（NMPA 批准用于非小细胞肺癌）、**厄洛替尼*（Erlotinib）**（FDA/NMPA 批准用于非小细胞肺癌）、**吉非替尼*（Gefitinib）**（FDA/NMPA 批准用于非小细胞肺癌）、**阿法替尼*（Afatinib）**（FDA/NMPA 批准用于非小细胞肺癌）、**达可替尼*（Dacomitinib）**（FDA/NMPA 批准用于非小细胞肺癌）、**奥希替尼*（Osimertinib）**（FDA/NMPA 批准用于非小细胞肺癌）；<br>EGFR 单抗：**西妥昔单抗*（Cetuximab）**（FDA/NMPA 批准用于结直肠癌；FDA 批准用于头颈部鳞状细胞癌）、**帕尼单抗（Panitumumab）**（FDA 批准用于结直肠癌）；<br>**曲妥珠单抗*（Trastuzumab）**（FDA/NMPA 批准用于乳腺癌）、**恩美曲妥珠单抗*（Ado-trastuzumab emtansine）**（FDA/NMPA 批准用于乳腺癌）、**维迪西妥单抗*（Disitamab）**（NMPA 批准用于胃癌、胃食管结合腺癌、膀胱尿路上皮癌）、**曲妥珠单抗+帕妥珠单抗+多西他赛（Trastuzumab+Pertuzumab+Docetaxel）**（FDA 批准用于乳腺癌）、**Trastuzumab deruxtecan**（FDA 批准用于乳腺癌、胃癌、胃食管结合腺癌）、**拉帕替尼+卡培他滨*（Lapatinib+Capecitabine）**（FDA/NMPA 批准用于乳腺癌）、**奈拉替尼+卡培他滨（Neratinib+Capecitabine）**（FDA 批准用于乳腺癌）、**Tucatinib+曲妥珠单抗+卡培他滨（Tucatinib+Trastuzumab+Capecitabine）**（FDA 批准用于乳腺癌）、**Margetuximab+化疗**（FDA 批准用于乳腺癌）、**伊尼妥单抗（Inetetamab）+化疗**（NMPA 批准用于乳腺癌）、**曲妥珠单抗+顺铂+卡培他滨*（Trastuzumab+Cisplatin+Capecitabine）**（FDA/NMPA 批准用于胃癌、胃食管结合腺癌）、**曲妥珠单抗+顺铂+氟尿嘧啶*（Trastuzumab+Cisplatin+5-Fluorouracil）**（FDA/NMPA 批准用于胃癌、胃食管结合腺癌）、**曲妥珠单抗+帕妥珠单抗+透明质酸酶+多西他赛（Trastuzumab+Pertuzumab+Hyaluronidase+Docetaxel）**（FDA 批准用于乳腺癌）、**曲妥珠单抗+帕妥珠单抗+透明质酸酶（Trastuzumab+Pertuzumab+Hyaluronidase）+化疗**（FDA 批准用于乳腺癌）、**曲妥珠单抗+帕妥珠单抗（Trastuzumab+Pertuzumab）+化疗***（FDA/NMPA 批准用于乳腺癌）、曲妥珠单抗+拉帕替尼（Trastuzumab+Lapatinib）（NCCN 指南推荐用于乳腺癌、结直肠癌）、曲妥珠单抗+长春瑞滨（Trastuzumab+Vinorelbine）（NCCN 指南推荐用于乳腺癌）	在乳腺癌中，HER2 扩增对曲妥珠单抗+帕妥珠单抗+透明质酸酶、曲妥珠单抗+帕妥珠单抗+多西他赛、曲妥珠单抗、恩美曲妥珠单抗、曲妥珠单抗+拉帕替尼、Trastuzumab deruxtecan、奈拉替尼、曲妥珠单抗+长春瑞滨、拉帕替尼+卡培他滨、卡铂+紫杉醇+曲妥珠单抗、奈拉替尼+卡培他滨、曲妥珠单抗+卡培他滨、Margetuximab+化疗[PMID: 33480963]、伊尼妥单抗+化疗、曲妥珠单抗+帕妥珠单抗+紫杉醇、他莫昔芬+曲妥珠单抗、氟维司群+曲妥珠单抗敏感[PMID: 29182361; PMID: 25524798; PMID: 24099077; PMID: 28592618; PMID: 22149875]；在非小细胞肺癌中，HER2 突变对恩美曲妥珠单抗[PMID: 29989854]和 Trastuzumab deruxtecan [J Clin Oncol. 2020; 38(suppl 15): abstr 9504]敏感。HER2 突变对埃克替尼、厄洛替尼、吉非替尼、阿法替尼、达可替尼、奥希替尼、西妥昔单抗、帕尼单抗耐药[PMID: 22325357; PMID: 22761469]。在胃癌中，HER2 扩增对曲妥珠单抗+顺铂+卡培他滨[PMID: 20728210]、曲妥珠单抗+顺铂+氟尿嘧啶[PMID: 20728210]、帕博利珠单抗+曲妥珠单抗+铂类+氟嘧啶[PMID: 33167735]敏感。

基因	检测内容	靶向药物	用药提示（仅供参考）
ERBB2 (HER2)	突变<br>扩增	卡铂+紫杉醇+曲妥珠单抗（Carboplatin+Paclitaxel+Trastuzumab）（NCCN 指南推荐用于子宫内膜癌、乳腺癌、子宫癌）、曲妥珠单抗+卡培他滨（Trastuzumab+Capecitabine）（NCCN 指南推荐用于乳腺癌）、他莫昔芬+曲妥珠单抗（NCCN 指南推荐用于乳腺癌）、氟维司群+曲妥珠单抗（NCCN 指南推荐用于乳腺癌）、曲妥珠单抗+帕妥珠单抗+紫杉醇（Trastuzumab+Pertuzumab+Paclitaxel）（NCCN 指南推荐用于乳腺癌）、曲妥珠单抗+化疗（Trastuzumab+Chemotherapy）（NCCN 指南推荐用于乳腺癌、胃腺癌）、帕博利珠单抗+曲妥珠单抗+铂类+氟嘧啶（Pembrolizumab+Trastuzumab+Platinum+Fluoropyrimidine）（NCCN 指南推荐用于胃腺癌）	
HRAS	突变	Tipifarnib（临床 II 期）	在头颈部鳞状细胞癌和转移性尿路上皮癌中，HRAS 突变对 Tipifarnib 敏感 [Cancer Discov. 9, 1637–1638 (2019); PMID: 32636318]。
KIT	突变	**伊马替尼*（Imatinib）**（FDA/NMPA 批准用于胃肠道间质瘤、系统性肥大细胞增多症等）、**培唑帕尼*（Pazopanib）**（FDA/NMPA 批准用于肾细胞癌；FDA 批准用于软组织肉瘤）、**普纳替尼（Ponatinib）**（FDA 批准用于慢性髓细胞白血病、急性淋巴细胞白血病）、**瑞戈非尼*（Regorafenib）**（FDA/NMPA 批准用于肝细胞癌、结直肠癌、胃肠道间质瘤）、**尼洛替尼*（Nilotinib）**（FDA/NMPA 批准用于慢性髓细胞白血病）、**达沙替尼*（Dasatinib）**（FDA/NMPA 批准用于慢性髓细胞白血病；FDA 批准用于急性淋巴细胞白血病）、**舒尼替尼*（Sunitinib）**（FDA/NMPA 批准用于胃肠道间质瘤）、**瑞派替尼*（Ripretinib）**（FDA/NMPA 批准用于胃肠道间质瘤）	胃肠道间质瘤中，KIT 11 号外显子突变对伊马替尼[PMID: 26687836]、培唑帕尼[PMID: 27068858]、普纳替尼[J Clin Oncol. 2015; 33(suppl 15): abstr 10535]、瑞戈非尼[PMID: 27371698; PMID: 22614970]、尼洛替尼[PMID: 25882987]和达沙替尼[PMID: 29315500]、舒尼替尼[PMID: 26772734]、瑞派替尼[PMID: 32511981]敏感，与 11 号外显子突变相比，9 号外显子突变对伊马替尼的敏感性中等[PMID: 14645423]；在皮肤黑色素瘤中，KIT 激活突变对伊马替尼敏感[PMID: 23775962; PMID: 21642685]。

**KRAS**	突变	EGFR 抑制剂：**厄洛替尼*（Erlotinib）**（FDA/NMPA 批准用于非小细胞肺癌）、**吉非替尼*（Gefitinib）**（FDA/NMPA 批准用于非小细胞肺癌）、**阿法替尼*（Afatinib）**（FDA/NMPA 批准用于非小细胞肺癌）、**达可替尼*（Dacomitinib）**（FDA/NMPA 批准用于非小细胞肺癌）、**奥希替尼*（Osimertinib）**(FDA/NMPA 批准用于非小细胞肺癌）、**埃克替尼*（Icotinib）**（NMPA 批准用于非小细胞肺癌）；<br>**Sotorasib**（FDA 批准用于非小细胞肺癌）；<br>索拉非尼+卡铂+紫杉醇（Sorafenib+Carboplatin+Paclitaxel）（临床 III 期）；<br>EGFR 单抗：**西妥昔单抗*（Cetuximab）**（FDA/NMPA 批准用于结直肠癌；FDA 批准用于头颈部鳞状细胞癌）、**帕尼单抗（Panitumumab）**（FDA 批准用于结直肠癌）	结直肠癌中，KRAS 野生型对 EGFR 抗体类药物西妥昔单抗和帕尼单抗敏感 [PMID: 18202412; PMID: 18316791]，12、13、61 密码子突变导致 KRAS 信号通路持续激活，可能无法从 EGFR 抗体类药物获益；肺癌中，12、13、61 密码子突变对 EGFR TKI（吉非替尼、厄洛替尼、埃克替尼、阿法替尼等）的敏感性降低[PMID: 18349398; PMID: 23401440; PMID: 18024870]；非小细胞肺癌中，KRAS G12C 突变的患者对 Sotorasib 敏感[PMID: 32955176]；黑色素瘤中，KRAS 扩增的患者对索拉非尼+卡铂+紫杉醇敏感[PMID: 26307133]。

基因	检测内容	靶向药物	用药提示（仅供参考）
MET	突变扩增	**克唑替尼*（Crizotinib）**（FDA/NMPA 批准用于非小细胞肺癌；FDA 批准用于间变性大细胞淋巴瘤）、**Capmatinib**（FDA 批准用于非小细胞肺癌）、**赛沃替尼**（**Savolitinib**）（NMPA 批准用于非小细胞肺癌）、**Tepotinib**（FDA 批准用于非小细胞肺癌）；<br>EGFR 抑制剂：**厄洛替尼*（Erlotinib）**（FDA/NMPA 批准用于非小细胞肺癌）、**吉非替尼*（Gefitinib）**（FDA/NMPA 批准用于非小细胞肺癌）、**阿法替尼*（Afatinib）**（FDA/NMPA 批准用于非小细胞肺癌）、**达可替尼*（Dacomitinib）**（FDA/NMPA 批准用于非小细胞肺癌）、**奥希替尼*（Osimertinib）**（FDA/NMPA 批准用于非小细胞肺癌）、**埃克替尼*（Icotinib）**（NMPA 批准用于非小细胞肺癌）	非小细胞肺癌中，高水平的 MET 扩增对克唑替尼[PMID: 21623265; J Clin Oncol. 2014; 32(suppl 15): abstr 8001]、Capmatinib [PMID: 32877583]敏感，MET 外显子 14 跳跃突变对 Capmatinib [PMID: 32877583]、赛沃替尼[J Clin Oncol. 2020; 38(suppl 15): abstr 9519]、克唑替尼[PMID: 31932802]、Tepotinib 敏感[PMID: 32469185]；并且 MET 扩增与 EGFR 突变的非小细胞肺癌患者的 EGFR 抑制剂（吉非替尼、厄洛替尼、埃克替尼、阿法替尼、达可替尼、奥希替尼）耐药相关[PMID: 29624806; PMID: 31131689; PMID: 27528220; PMID: 29571987]。
NRAS	突变	贝美替尼（Binimetinib）（NCCN 指南推荐用于皮肤黑色素瘤）；<br>EGFR 单抗：**西妥昔单抗*（Cetuximab）**（FDA/NMPA 批准用于结直肠癌；FDA 批准用于头颈部鳞状细胞癌）、**帕尼单抗（Panitumumab）**（FDA 批准用于结直肠癌）	结直肠癌中，NRAS 野生型对西妥昔单抗[PMID: 30463680]、帕尼单抗（EGFR 单抗）[PMID: 29587749; PMID: 29737864]敏感性增加；黑色素瘤中，NRAS 突变的患者对贝美替尼敏感[PMID: 28284557]。
PDGFRA	突变	**伊马替尼*（Imatinib）**（FDA/NMPA 批准用于胃肠道间质瘤、慢性髓细胞白血病等）；<br>**达沙替尼*（Dasatinib）**（FDA/NMPA 批准用于慢性髓细胞白血病；FDA 批准用于急性淋巴细胞白血病）；<br>**阿伐替尼*（Avapritinib）**（FDA/NMPA 批准用于胃肠道间质瘤；FDA 批准用于系统性肥大细胞增多症）	胃肠道间质瘤中，PDGFRA 突变（除 D842V）对伊马替尼敏感[PMID: 12181401]，PDGFRA D842V 对达沙替尼敏感[PMID: 29315500; PMID: 18794084]，PDGFRA 突变 18 号外显子对阿伐替尼敏感[PMID: 33465704]，PDGFRA D842V 对伊马替尼产生耐药[PMID: 22718859]。
PIK3CA	突变	**Alpelisib+氟维司群（Alpelisib+Fulvestrant）**（FDA 批准用于乳腺癌）	乳腺癌中，PIK3CA 激活突变（E542K、E545K、E545Q、H1047L、H1047R 等）可能对 Alpelisib+氟维司群敏感[PMID：31091374]。
PTEN	突变	**替西罗莫司（Temsirolimus）**（FDA 批准用于肾细胞腺癌）	非霍奇金淋巴瘤和多发性骨髓瘤中，PTEN 失活突变对替西罗莫司敏感[PMID: 16916489]。

基因	检测内容	靶向药物	用药提示（仅供参考）
RET	突变融合	**普拉替尼*（Pralsetinib）**（FDA/NMPA 批准用于非小细胞肺癌、甲状腺癌、甲状腺髓样癌）；<br>**Selpercatinib**（FDA 批准用于甲状腺癌、非小细胞肺癌、甲状腺髓样癌）；<br>**凡德他尼（Vandetanib）**（FDA 批准用于甲状腺髓样癌）；<br>**卡博替尼（Cabozantinib）**（FDA 批准用于甲状腺髓样癌、肾癌、肝细胞癌）；<br>**仑伐替尼*（Lenvatinib）**（FDA/NMPA 批准用于肝细胞癌、甲状腺癌）	甲状腺癌中，RET 融合和突变对 Selpercatinib 敏感[PMID: 33239432]，RET M918T 对卡博替尼敏感[PMID: 27525386]；肺癌中，RET 融合对仑伐替尼敏感[PMID: 31710864]；非小细胞肺癌中，RET 融合和突变对 Selpercatinib 敏感[PMID: 33239432]、RET 融合对普拉替尼敏感[PMID: 33771190]，临床试验研究表明，RET 融合对卡博替尼[PMID: 27825636]和凡德他尼[PMID: 27825616]敏感；凡德他尼是多靶点激酶抑制剂，RET 是靶点之一[PMID: 27825616]。
ROS1	突变融合	**克唑替尼*（Crizotinib）**（FDA/NMPA 批准用于非小细胞肺癌；FDA 批准用于间变性大细胞淋巴瘤）；<br>**塞瑞替尼*（Ceritinib）**（FDA/NMPA 批准用于非小细胞肺癌）；<br>**恩曲替尼（Entrectinib）**（FDA 批准用于实体瘤、非小细胞肺癌）；<br>**劳拉替尼（Lorlatinib）**（FDA 批准用于非小细胞肺癌）	非小细胞肺癌中，ROS1 融合对克唑替尼（ALK/MET/ROS1 抑制剂）[PMID: 30980071; PMID: 29596029]、塞瑞替尼[PMID: 28520527]、恩曲替尼[PMID: 31838015]、劳拉替尼[PMID: 31669155]敏感，ROS1 融合与 EGFR TKI 的原发性耐药有关[PMID: 30568455; PMID: 31124056]。
TSC1	突变	mTOR 抑制剂：**依维莫司* (Everolimus)**（FDA/NMPA 批准用于肾癌、神经内分泌癌、脂肪肉瘤等）	膀胱癌中，TSC1 E636 移码突变对 mTOR 抑制剂依维莫司敏感[PMID: 22923433]。

检测基因列表

A

ABCB1	*ABCC2*	*ABCC4*	*ABCG2*	*ABL1*	*ABRAXAS1*	*ALK*
APC	*AR*	*ARID1B*	*ASXL1*	*ATM*	*ATR*	*AURKA*

B

BAP1	*BARD1*	*BCL11A*	*BCL11B*	*BCORL1*	*BLM*	*BRAF*
BRCA1	*BRCA2*	*BRD3*	*BRIP1*			

C

C8orf34	*CASP3*	*CASP9*	*CCDC6*	*CDH1*	*CDK1*	*CDK12*
CDK7	*CDK8*	*CDK9*	*CDKN1C*	*CHEK1*	*CHEK2*	*CREBBP*
CTNNA1	*CUL3*	*CXCL8*	*CXCR4*	*CYP19A1*	*CYP1B1*	*CYP2B6*
CYP2C8						

D

DDIT3	*DDR1*	*DHFR*	*DPYD*			

E

EGFR	*EMSY*	*EP300*	*EPCAM*	*EPHA5*	*EPHA6*	*EPHA7*
ERBB2	*ERCC1*	*ERCC2*	*ERCC3*	*ERCC4*	*ESR1*	*ESR2*
EWSR1	*EZH2*					

F

FANCA	*FANCC*	*FANCD2*	*FANCE*	*FANCF*	*FANCG*	*FANCI*
FANCL	*FANCM*	*FGFR1*	*FGFR2*	*FGFR3*	*FLT3*	

G

GATA1	*GATA4*	*GEN1*	*GGH*	*GSTP1*		

H

H1-2	*H1-5*	*HDAC2*	*HRAS*			

I

IDH1	*IDH2*	*IGF2R*				

K

KDR	*KIT*	*KMT2A*	*KMT2B*	*KMT2C*	*KMT2D*	*KRAS*

M

MAP2K4	*MAP3K1*	*MAP4K1*	*MAP4K4*	*MAPK14*	*MET*	*MLH1*
MLH3	*MMP14*	*MMP2*	*MMP9*	*MRE11*	*MSH2*	*MSH6*
MTHFR	*MTR*	*MUTYH*				

N

NBN	*NCOR1*	*NCOR2*	*NF1*	*NOTCH3*	*NOTCH4*	*NQO1*
NRAS	*NTRK1*	*NTRK2*	*NTRK3*			

P

PALB2	*PARP1*	*PARP2*	*PARP3*	*PARP4*	*PDGFRA*	*PDGFRB*
PIK3C3	*PIK3CA*	*PIK3CD*	*PML*	*PMS1*	*PMS2*	*POLD1*
POLD2	*POLE*	*POLE2*	*PPM1D*	*PPP2R2A*	*PRKDC*	*PTEN*

R

RAD50	*RAD51*	*RAD51B*	*RAD51C*	*RAD51D*	*RAD52*	*RAD54B*
RAD54L	*RB1*	*RECQL4*	*RET*	*ROS1*	*RPA1*	*RRM1*

S

SEMA3C	*SLC19A1*	*SLC22A2*	*SLC28A1*	*SLCO1B1*	*SLX4*	*SOD2*
STAT3	*STAT4*	*STK11*				

T

TGFBR1	*TGFBR2*	*TNFRSF14*	*TNFRSF17*	*TP53*	*TPMT*	*TSC1*
TSC2						

U

UGT1A1	*UMPS*					

W

WRN						

X

XPC	*XRCC1*	*XRCC2*				

Z

ZBTB16	*ZNF217*					

检测流程

1

样本收集

样本采集、寄送及接收

2

实验过程

DNA 提取、质量控制

文库构建、质量控制

基因测序

3

数据分析

预处理、质量控制

对比、质量控制

注解、质量控制

过滤检测

4

医学解读

医学报告解读

报告审核

5

报告交付

参考文献

Solomon BJ, Mok T, Kim DW, et al. First-line crizotinib versus chemotherapy in ALK-positive lung cancer. N Engl J Med. 2014;371(23):2167-77. [PMID: 25470694]

Soria JC, Tan DSW, Chiari R, et al. First-line ceritinib versus platinum-based chemotherapy in advanced ALK-rearranged non-small-cell lung cancer (ASCEND-4): a randomised, open-label, phase 3 study. Lancet. 2017;389(10072):917-929. [PMID: 28126333]

Peters S, Camidge DR, Shaw AT, et al. Alectinib versus crizotinib in untreated ALK-positive non-small-cell lung cancer. N Engl J Med. 2017;377(9):829-838. [PMID: 28586279]

Camidge DR, Kim HR, Ahn MJ, et al. Brigatinib versus crizotinib in ALK-positive non-small-cell lung cancer. N Engl J Med. 2018, 379(21):2027-2039. [PMID: 30280657]

Shaw AT, Bauer TM, de Marinis F, et al. First-line lorlatinib or crizotinib in advanced ALK-positive lung cancer. N Engl J Med. 2020;383(21):2018-2029. [PMID: 33207094]

Horn L, Infante JR, Reckamp KL, et al. Ensartinib (X-396) in ALK-positive non-small cell lung cancer: Results from a first-in-human phase I/II, multicenter study. Clin Cancer Res. 2018;24(12):2771-2779. [PMID: 29563138]

Shaw AT, Yeap BY, Mino-Kenudson M, et al. Clinical features and outcome of patients with non-small-cell lung cancer who harbor EML4-ALK. J Clin Oncol. 2009;27(26):4247-53. [PMID: 19667264]

Katayama R, Shaw AT, Khan TM, et al. Mechanisms of acquired crizotinib resistance in ALK-rearranged lung Cancers. Sci Transl Med. 2012;4(120):120ra17. [PMID: 22277784]

Friboulet L, Li N, Katayama R, et al. The ALK inhibitor ceritinib overcomes crizotinib resistance in non-small cell lung cancer. Cancer Discov. 2014;4(6):662-673. [PMID: 24675041]

Song Z, Wang M, Zhang A. Alectinib: a novel second generation anaplastic lymphoma kinase (ALK) inhibitor for overcoming clinically-acquired resistance. Acta Pharm Sin B. 2015;5(1):34-7. [PMID: 26579422]

Butrynski JE, D'Adamo DR, Hornick JL, et al. Crizotinib in ALK-rearranged inflammatory myofibroblastic tumor. N Engl J Med. 2010;363(18):1727-33. [PMID: 20979472]

Shaw AT, Kim DW, Mehra R, et al. Ceritinib in ALK-rearranged non-small-cell lung cancer. N Engl J Med. 2014;370(13):1189-97. [PMID: 24670165]

Gettinger SN, Bazhenova LA, Langer CJ, et al. Activity and safety of brigatinib in ALK-rearranged non-small-cell lung cancer and other malignancies: a single-arm, open-label, phase 1/2 trial. Lancet Oncol. 2016;17(12):1683-1696. [PMID: 27836716]

Sekimizu M, Osumi T, Fukano R, et al. A phase I/II study of crizotinib for recurrent or refractory anaplastic lymphoma kinase-positive anaplastic large cell lymphoma and a phase I Study of crizotinib for recurrent or refractory neuroblastoma: Study protocol for a multicenter single-arm open-label trial. Acta Med Okayama. 2018;72(4):431-436. [PMID: 30140094]

Hainsworth JD, Meric-Bernstam F, Swanton C, et al. Targeted therapy for advanced solid tumors on the basis of molecular profiles: Results from MyPathway, an open-label, phase IIa multiple basket study. J Clin Oncol. 2018;36(6):536-542. [PMID: 29320312]

Planchard D, Kim TM, Mazieres J, et al. Dabrafenib in patients with BRAF(V600E)-positive advanced non-small-cell lung cancer: a single-arm, multicentre, open-label, phase 2 trial. Lancet Oncol. 2016;17(5):642-50. [PMID: 27080216]

Kefford R, Miller WH, Tan DSW, et al. Preliminary results from a phase Ib/II, open-label, dose-escalation study of the oral BRAF inhibitor LGX818 in combination with the oral MEK1/2 inhibitor MEK162 in BRAF V600-dependent advanced solid tumors. J Clin Oncol. 2013;31(suppl 15): abstr 9029.

Dummer R, Ascierto PA, Gogas HJ, et al. Encorafenib plus binimetinib versus vemurafenib or encorafenib in patients with BRAF-mutant melanoma (COLUMBUS): a multicentre, open-label, randomised phase 3 trial. Lancet Oncol. 2018;19(5):603-615. [PMID: 29573941]

Larkin J, Ascierto PA, Dréno B, et al. Combined vemurafenib and cobimetinib in BRAF-mutated melanoma. N Engl J Med. 2014;371(20):1867-76. [PMID: 25265494]

Ascierto PA, McArthur GA, Dréno B, et al. Cobimetinib combined with vemurafenib in advanced BRAF(V600)-mutant melanoma (coBRIM): updated efficacy results from a randomised, double-blind, phase 3 trial. Lancet Oncol. 2016;17(9):1248-60. [PMID: 27480103]

Fangusaro J, Onar-Thomas A, Poussaint TY, et al. Selumetinib in paediatric patients with BRAFaberrant or neurofibromatosis type 1-associated recurrent, refractory, or progressive low-grade glioma: a multicentre, phase 2 trial. Lancet Oncol 2019;20:1011-1022. [PMID: 31151904]

Long GV, Weber JS, Infante JR, et al. Overall survival and durable responses in patients with BRAF V600-mutant metastatic melanoma receiving dabrafenib combined with trametinib. J Clin Oncol. 2016;34(8):871-8. [PMID: 26811525]

Gutzmer R, Stroyakovskiy D, Gogas H, et al. Atezolizumab, vemurafenib, and cobimetinib as first-line treatment for unresectable advanced BRAF V600 mutation-positive melanoma (IMspire150): Primary analysis of the randomised, double-blind, placebo-controlled, phase 3 trial. Lancet. 2020;395(10240):1835-1844. [PMID: 32534646]

Van Cutsem E, Hujberts S, Grothey A, et al. Binimetinib, encorafenib, and cetuximab triplet therapy for patients with BRAF V600E-mutant metastatic colorectal cancer: Safety lead-in results from the phase III BEACON colorectal cancer study. J Clin Oncol. 2019;37(17):1460-1469. [PMID: 30892987]

Kopetz S, Grothey A, Yaeger R, et al. Encorafenib, binimetinib, and cetuximab in BRAF V600E-mutated colorectal cancer. N Engl J Med. 2019;381(17):1632-1643. [PMID: 31566309]

Kopetz S, Grothey A, Van CutsemE, et al. Encorafenib plus cetuximab with or without binimetinib for BRAF V600E-mutant metastatic colorectal cancer: Quality-of-life results from a randomized, three-arm, phase III study versus the choice of either irinotecan or FOLFIRI plus cetuximab (BEACON CRC). J Clin Oncol. 2020;38(suppl 4): abstr 8.

Kopetz S, Mcdonough S L, Morris V K, et al. Randomized trial of Irinotecan and Cetuximab with or without Vemurafenib in BRAF-mutant metastatic colorectal cancer (SWOG 1406).[J]. Journal of Clinical Oncology, 2017, 35(4_suppl):520-520. [PMID: 33356422]

Hyman DM, Puzanov I, Subbiah V, et al. Vemurafenib in multiple nonmelanoma cancers with BRAF V600 mutations. N Engl J Med. 2015;373(8):726-36. [PMID: 26287849]

Pietrantonio F, Petrelli F, Coinu A, et al. Predictive role of BRAF mutations in patients with advanced colorectal cancer receiving cetuximab and panitumumab: a meta-analysis. Eur J Cancer. 2015;51(5):587-94. [PMID: 25673558]

Long GV, Flaherty KT, Stroyakovskiy D, et al. Dabrafenib plus trametinib versus dabrafenib monotherapy in patients with metastatic BRAF V600E/K-mutant

melanoma: long-term survival and safety analysis of a phase 3 study. Ann Oncol. 2017;28(7):1631-1639. [PMID: 28475671]

Planchard D, Smit EF, Groen HJM, et al. Dabrafenib plus trametinib in patients with previously untreated BRAF V600E-mutant metastatic non-small-cell lung cancer: an open-label, phase 2 trial. Lancet Oncol. 2017;18: 1307-1316. [PMID: 28919011]

Nakagawa K, Garon EB, Seto T, et al. Ramucirumab plus erlotinib in patients with untreated, EGFR-mutated, advanced non-small-cell lung cancer (RELAY): a randomised, double-blind, placebo-controlled, phase 3 trial. Lancet Oncol. 2019;20(12):1655-1669. [PMID: 31591063]

Soria JC, Ohe Y, Vansteenkiste J, et al. Osimertinib in untreated EGFR-mutated advanced non-small-cell lung cancer. N Engl J Med. 2018;378(2):113-125. [PMID: 29151359]

Yang JCH, Camidge DR, Yang CT, et al. Safety, efficacy, and pharmacokinetics of almonertinib (HS-10296) in pretreated patients with EGFR-mutated advanced NSCLC: A multicenter, open-label, phase 1 trial. J Thorac Oncol. 2020;15(12):1907-1918. [PMID: 32916310]

Shi Y, Hu X, Zhang S, et al. Efficacy and safety of alflutinib (AST2818) in patients with T790M mutation-positive NSCLC: A phase IIb multicenter single-arm study. J Clin Oncol. 2020;38(suppl 15): abstr 9602.

Robichaux JP, Elamin YY, Tan Z, et al. Mechanisms and clinical activity of an EGFR and HER2 exon 20-selective kinase inhibitor in non-small cell lung cancer. Nat Med. 2018;24(5):638-646. [PMID: 29686424]

[No authors listed]. Poziotinib shows promise for rare lung cancer. Cancer Discov. 2018;8(1):OF4. doi: 10.1158/2159-8290. [PMID: 29162564]

Yun J, Lee SH, Kim SY, et al. Antitumor Activity of Amivantamab (JNJ-61186372), an EGFR-MET Bispecific Antibody, in Diverse Models of EGFR Exon 20 Insertion-Driven NSCLC[J]. Cancer Discov. 2020, 10(8):1194-1209. [PMID: 32414908]

Heymach JV, Lockwood SJ, Herbst RS, et al. EGFR biomarkers predict benefit from vandetanib in combination with docetaxel in a randomized phase III study of second-line treatment of patients with advanced non-small cell lung cancer. Ann Oncol. 2014;25(10):1941-1948. [PMID: 25057173]

Saito H, Fukuhara T, Furuya N, et al. Erlotinib plus bevacizumab versus erlotinib alone in patients with EGFR-positive advanced non-squamous non-small-cell lung cancer (NEJ026): interim analysis of an open-label, randomised, multicentre, phase 3 trial. Lancet Oncol. 2019;20(5):625-635. [PMID: 30975627]

Sartore-Bianchi A, Moroni M, Veronese S, et al. Epidermal growth factor receptor gene copy number and clinical outcome of metastatic colorectal cancer treated with panitumumab. J Clin Oncol. 2007;25(22):3238-45. [PMID: 17664472]

Lv S, Teugels E, Sadones J, et al. Correlation of EGFR, IDH1 and PTEN status with the outcome of patients with recurrent glioblastoma treated in a phase II clinical trial with the EGFR-blocking monoclonal antibody cetuximab. Int J Oncol. 2012;41(3):1029-35. [PMID: 22752145]

Genova C, Socinski MA, Hozak RR, et al. EGFR gene copy number by FISH may predict outcome of necitumumab in squamous lung carcinomas: Analysis from the SQUIRE study. J Thorac Oncol. 2018;13(2): 228-236. [PMID: 29158193]

Papadopoulos KP, Tolcher AW, Patnaik A, et al. Phase 1, first-in-human study of ARQ 087, an oral pan-Fibroblast Growth Factor Receptor (FGFR) inhibitor, in patients (pts) with advanced solid tumors. J Clin Oncol. 2015;33(suppl 15): abstr 2545.

Nogova L, Sequist LV, Perez Garcia JM, et al. Evaluation of BGJ398, a fibroblast growth factor receptor 1-3 kinase inhibitor, in patients with advanced solid tumors harboring genetic alterations in fibroblast growth factor receptors: Results of a global phase I, dose-escalation and dose-expansion study. J Clin Oncol. 2017;35(2):157-165. [PMID: 27870574]

André F, Bachelot T, Campone M, et al. Targeting FGFR with dovitinib (TKI258): preclinical and clinical data in breast cancer. Clin Cancer Res. 2013;19(13):3693-702. [PMID: 23658459]

Abou-Alfa GK, Sahai V, Hollebecque A, et al. Pemigatinib for previously treated, locally advanced or metastatic cholangiocarcinoma: a multicentre, open-label, phase 2 study. Lancet Oncol. 2020;21(5):671-684. [PMID: 32203698]

Javle M, Lowery M, Shroff RT, et al. Phase II study of BGJ398 in patients with FGFR-altered advanced cholangiocarcinoma. J Clin Oncol. 2018;36(3):276-282. [PMID: 29182496]

Pearson A, Smyth E, Babina IS, et al. High-level clonal FGFR amplification and response to FGFR inhibition in a translational clinical trial. Cancer Discov. 2016;6(8):838-851. [PMID: 27179038]

Loriot Y, Necchi A, Park SH, et al. Erdafitinib in locally advanced or metastatic urothelial carcinoma. N Engl J Med. 2019;381(4):338-348. [PMID: 31340094]

Fabi A, Giannarelli D, Moscetti L, et al. Ado-trastuzumab emtansine (T-DM1) in HER2+ advanced breast cancer patients: does pretreatment with pertuzumab matter. Future Oncol. 2017;13(30):2791-2797. [PMID: 29182361]

Finn RS, Crown JP, Lang I, et al. The cyclin-dependent kinase 4/6 inhibitor Palbociclib in combination with Letrozole versus Letrozole alone as first-line treatment of oestrogen receptor-positive, HER2-negative, advanced breast cancer (PALOMA-1/TRIO-18): a randomised phase 2 study. Lancet Oncol. 2015;16(1):25-35. [PMID: 25524798]

Wolff AC, Hammond ME, Hicks DG, et al. Recommendations for human epidermal growth factor receptor 2 testing in breast cancer: American Society of Clinical Oncology/College of American Pathologists clinical practice guideline update. Arch Pathol Lab Med. 2013;31(31):3997. [PMID: 24099077]

Andersson M, López-Vega JM, Petit T, et al. Efficacy and safety of pertuzumab and trastuzumab administered in a single infusion bag, followed by vinorelbine: VELVET cohort 2 final results. Oncologist. 2017;22(10):1160-1168. [PMID: 28592618]

José B, Javier C, Sung-Bae K, et al. Pertuzumab plus trastuzumab plus docetaxel for metastatic breast cancer. N Engl J Med. 2012;366(2):109-19. [PMID: 22149875]

Li BT, Shen R, Buonocore D, et al. Ado-trastuzumab emtansine for patients with HER2-mutant lung cancers: Results from a phase II basket trial. J Clin Oncol. 2018;36(24):2532-2537. [PMID: 29989854]

Smit EF, Nakagawa K, Nagasaka M, et al. Trastuzumab deruxtecan (T-DXd; DS-8201) in patients with HER2-mutated metastatic non-small cell lung cancer (NSCLC): Interim results of DESTINY-Lung01. J Clin Oncol. 2020;38(suppl 15): abstr 9504.

De Grève J, Teugels E, Geers C, et al. Clinical activity of afatinib (BIBW 2992) in patients with lung adenocarcinoma with mutations in the kinase domain of HER2/neu. Lung Cancer. 2012;76(1): 123-127. [PMID: 22325357]

Arcila ME, Chaft JE, Nafa K, et al. Prevalence, clinicopathologic associations, and molecular spectrum of ERBB2 (HER2) tyrosine kinase mutations in lung adenocarcinomas. Clin Cancer Res. 2012; 18(18): 4910-4918. [PMID: 22761469]

Bang YJ, Van Cutsem E, Feyereislova A, et al. Trastuzumab in combination with chemotherapy versus chemotherapy alone for treatment of HER2-positive advanced gastric or gastro-oesophageal junction cancer (ToGA): a phase 3, open-label, randomised controlled trial. Lancet. 2010;376(9742):687-97. [PMID: 20728210]

[No authors listed] Tipifarnib targets HRAS-mutant cancers. Cancer Discov. 2019;9:1637–1638

Lee HW, Sa JK, Gualberto A, et al. A phase II trial of tipifarnib for patients with previously treated, metastatic urothelial carcinoma harboring HRAS mutations. Clin Cancer Res. 2020; 26(19): 5113-5119. [PMID: 32636318]

Patrikidou A, Domont J, Chabaud S, et al. Long-term outcome of molecular subgroups of GIST patients treated with standard-dose imatinib in the BFR14 trial of the French Sarcoma Group. Eur J Cancer. 2016;52:173-80. [PMID: 26687836]

Mir O, Cropet C, Toulmonde M, et al. Pazopanib plus best supportive care versus best supportive care alone in advanced gastrointestinal stromal tumours resistant to imatinib and sunitinib (PAZOGIST): A randomised, multicentre, open-label phase 2 trial. Lancet Oncol. 2016;17(5):632-41. [PMID: 27068858]

Heinrich MC, Mehren Mv, Demetri GD, et al. Ponatinib efficacy and safety in patients (pts) with advanced gastrointestinal stromal tumors (GIST) after tyrosine kinase inhibitor (TKI) failure: Results from a phase 2 study. J Clin Oncol. 2015;33 (suppl 15): abstr 10535.

Ben-Ami E, Barysauskas CM, von Mehren M, et al. Long-term follow-up results of the multicenter phase II trial of regorafenib in patients with metastatic and/or unresectable GI stromal tumor after failure of standard tyrosine kinase inhibitor therapy. Ann Oncol. 2016;27(9):1794-9. [PMID: 27371698]

George S, Wang Q, Heinrich MC, et al. Efficacy and safety of regorafenib in patients with metastatic and/or unresectable GI stromal tumor after failure of imatinib and sunitinib: a multicenter phase II trial. J Clin Oncol. 2012;30(19):2401-7. [PMID: 22614970]

Blay JY, Shen L, Kang YK, et al. Nilotinib versus imatinib as first-line therapy for patients with unresectable or metastatic gastrointestinal stromal tumours (ENESTg1): A randomised phase 3 trial. Lancet Oncol. 2015 May;16(5):550-60. [PMID: 25882987]

Montemurro M, Cioffi A, Dômont J, et al. Long-term outcome of dasatinib first-line treatment in gastrointestinal stromal tumor: A multicenter, 2-stage phase 2 trial (Swiss Group for Clinical Cancer Research 56/07). Cancer. 2018;124(7): 1449-1454. [PMID: 29315500]

Heinrich MC, Corless CL, Demetri GD, et al. Kinase mutations and imatinib response in patients with metastatic gastrointestinal stromal tumor. J Clin Oncol. 2003;21(23):4342-9. [PMID: 14645423]

Hodi FS, Corless CL, Giobbie-Hurder A, et al. Imatinib for melanomas harboring mutationally activated or amplified KIT arising on mucosal, acral, and chronically sun-damaged skin. J Clin Oncol. 2013;31(26):3182-90. [PMID: 23775962]

Carvajal RD, Antonescu CR, Wolchok JD, et al. KIT as a therapeutic target in metastatic melanoma. JAMA. 2011;305(22):2327-34. [PMID: 21642685]

Lièvre A, Bachet J-B , Boige V, et al. KRAS mutations as an independent prognostic factor in patients with advanced colorectal cancer treated with cetuximab. J Clin Oncol. 2008;26(3):374-9. [PMID: 18202412]

Amado RG , Wolf M, Peeters M, et al. Wild-type KRAS is required for panitumumab efficacy in patients with metastatic colorectal cancer. J Clin Oncol. 2008;26(10):1626-34. [PMID: 18316791]

Miller VA, Riely GJ, Zakowski MF, et al. Molecular characteristics of bronchioloalveolar carcinoma and adenocarcinoma, bronchioloalveolar carcinoma subtype, predict response to erlotinib. J Clin Oncol. 2008;26(9):1472-8. [PMID: 18349398]

Roberts PJ , Stinchcombe TE. KRAS mutation: Should we test for it, and does it matter? J Clin Oncol. 2013;31(8):1112-21. [PMID: 23401440]

Tsao MS, Aviel-Ronen S, Ding K, et al. Prognostic and predictive importance of p53 and RAS for adjuvant chemotherapy in non small-cell lung cancer. J Clin Oncol. 2007;25(33):5240-7. [PMID: 18024870]

Hong DS, Fakih MG, Strickler JH, et al. KRAS G12C Inhibition with Sotorasib in Advanced Solid Tumors[J]. N Engl J Med. 2020, 24;383(13):1207-1217. [PMID: 32955176]

Wilson MA, Zhao F, Khare S, et al. Copy number changes are associated with response to treatment with carboplatin, paclitaxel, and sorafenib in melanoma. Clin Cancer Res. 2016;22(2):374-82. [PMID: 26307133]

Ou SH, Kwak EL, Siwak-Tapp C, Dy J, et al. Activity of crizotinib (PF02341066), a dual mesenchymal-epithelial transition (MET) and anaplastic lymphoma kinase (ALK) inhibitor, in a non-small cell lung cancer patient with de novo MET amplification. J Thorac Oncol. 2011;6(5):942-6. [PMID: 21623265]

Camidge DR, Ou S-HI, Shapiro G, et al. Efficacy and safety of crizotinib in patients with advanced c-MET-amplified non-small cell lung cancer (NSCLC). J Clin Oncol. 2014;32(suppl 15): abstr 8001.

Wolf J, Seto T, Han JY, Reguart N, et al. Capmatinib in *MET* exon 14-mutated or *MET*-amplified non-small-cell lung cancer. N Engl J Med. 2020;383(10):944-957. [PMID: 32877583]

Lu S et al., Phase II study of savolitinib in patients with pulmonary sarcomatoid carcinoma and other types of non-small cell lung cancer harboring MET exon 14 skipping mutations. J Clin Oncol. 2020;38(suppl 15): abstr 9519.

Drilon A, Clark JW, Weiss J, Ou SI, et al. Antitumor activity of crizotinib in lung cancers harboring a MET exon 14 alteration. Nat Med. 2020;26(1):47-51. [PMID: 31932802]

Paik PK, Felip E, Veillon R, et al. Tepotinib in non-small-cell lung cancer with *MET* exon 14 skipping mutations. N Engl J Med. 2020;383(10):931-943. [PMID: 32469185]

Feng W, Xie Q, Liu S, et al. Krüppel-like factor 4 promotes c-Met amplification-mediated gefitinib resistance in non-small-cell lung cancer. Cancer Sci. 2018; 109(6):1775-1786. [PMID: 29624806]

Li JW, Cao SH, Xu JL, et al. De novo MET amplification promotes intrinsic resistance to first-generation EGFR tyrosine kinase inhibitors. Cancer Biol Ther. 2019;20(9):1183-1186. [PMID: 31131689]

Jin Y, Shao Y, Shi X, et al. Mutational profiling of non-small-cell lung cancer patients resistant to first-generation EGFR tyrosine kinase inhibitors using next generation sequencing. Oncotarget. 2016;7(38):61755-61763. [PMID: 27528220]

Wang Y, Li L, Han R, et al. Clinical analysis by next-generation sequencing for NSCLC patients with MET amplification resistant to osimertinib. Lung Cancer. 2018;118:105-110. [PMID: 29571987]

Shapiro JD, Thavaneswaran S, Underhill CR, et al. Cetuximab alone or with irinotecan for resistant KRAS-, NRAS-, BRAF- and PIK3CA-wild-type metastatic colorectal cancer: The AGITG randomized phase II ICECREAM study. Clin Colorectal Cancer. 2018;17(4):313-319. [PMID: 30463680]

Geredeli C, Yasar N. FOLFIRI plus panitumumab in the treatment of wild-type KRAS and wild-type NRAS metastatic colorectal cancer. World J Surg Oncol. 2018;16(1):67. [PMID: 29587749]

McGregor M, Price TJ. Panitumumab in the treatment of metastatic colorectal cancer, including wild-type RAS, KRAS and NRAS mCRC. Future Oncol. 2018;14(24):2437-2459. [PMID: 29737864]

Dummer R, Schadendorf D, Ascierto PA, et al. Binimetinib versus dacarbazine in patients with advanced NRAS-mutant melanoma (NEMO): a multicentre, open-label, randomised, phase 3 trial. Lancet Oncol. 2017;18(4):435-445. [PMID: 28284557]

Demetri GD, von Mehren M, Blanke CD, et al. Efficacy and safety of imatinib mesylate in advanced gastrointestinal stromal tumors. N Engl J Med.

2002;347(7):472-80. [PMID: 12181401]

Dewaele B, Wasag B, Cools J, et al. Activity of dasatinib, a dual SRC/ABL kinase inhibitor, and IPI-504, a heat shock protein 90 inhibitor, against gastrointestinal stromal tumor-associated PDGFR AD842V mutation. Clin Cancer Res. 2008;14(18):5749-58. [PMID: 18794084]

Jones RL, Serrano C, von Mehren M, et al. Avapritinib in unresectable or metastatic PDGFRA D842V-mutant gastrointestinal stromal tumours: Long-term efficacy and safety data from the NAVIGATOR phase I trial. Eur J Cancer. 2021;145:132-142. [PMID: 33465704]

Cassier PA, Fumagalli E, Rutkowski P, et al. Outcome of patients with platelet-derived growth factor receptor alpha-mutated gastrointestinal stromal tumors in the tyrosine kinase inhibitor era. Clin Cancer Res. 2012;18(16):4458-64. [PMID: 22718859]

André F, Ciruelos E, Rubovszky G, et al. Alpelisib for PIK3CA-mutated, hormone receptor-positive advanced breast cancer. N Engl J Med. 2019;380(20):1929-1940. [PMID: 31091374]

Witzig T E, Kaufmann S H. Inhibition of the phosphatidylinositol 3-kinase/mammalian target of rapamycin pathway in hematologic malignancies[J]. Current Treatment Options in Oncology, 2006, 7(4):285-294. [PMID: 16916489]

Bradford D, Larkins E, Mushti SL, et al. FDA approval summary: Selpercatinib for the treatment of lung and thyroid cancers with RET gene mutations or fusions. Clin Cancer Res. 2021;27(8):2130-2135. [PMID: 33239432]

Drusbosky LM, Rodriguez E, Dawar R, et al. Therapeutic strategies in RET gene rearranged non-small cell lung cancer. J Hematol Oncol. 2021;14(1):50. [PMID: 33771190]

Sherman SI, Clary DO, Elisei R, et al. Correlative analyses of RET and RAS mutations in a phase 3 trial of cabozantinib in patients with progressive, metastatic medullary thyroid cancer. Cancer. 2016;122(24):3856-3864. [PMID: 27525386]

Hida T, Velcheti V, Reckamp KL, et al. A phase 2 study of lenvatinib in patients with RET fusion-positive lung adenocarcinoma. Lung Cancer. 2019;138:124-130. [PMID: 31710864]

Drilon A, Rekhtman N, Arcila M, et al. Cabozantinib in patients with advanced RET-rearranged non-small-cell lung cancer: an open-label, single-centre, phase 2, single-arm trial. Lancet Oncol. 2016;17(12):1653-1660. [PMID: 27825636]

Yoh K, Seto T, Satouchi M, et al. Vandetanib in patients with previously treated RET-rearranged advanced non-small-cell lung cancer (LURET): an open-label, multicentre phase 2 trial. Lancet Respir Med. 2017;5(1):42-50. [PMID: 27825616]

Shaw AT, Riely GJ, Bang YJ, et al. Crizotinib in ROS1-rearranged advanced non-small-cell lung cancer (NSCLC): updated results, including overall survival, from PROFILE 1001. Ann Oncol. 2019;30(7):1121-1126. [PMID: 30980071]

Wu YL, Yang JC, Kim DW, et al. Phase II study of crizotinib in east asian patients with ROS1-positive advanced non-small-cell lung cancer. J Clin Oncol. 2018;36(14):1405-1411. [PMID: 29596029]

Lim SM, Kim HR, Lee JS, et al. Open-label, multicenter, phase II study of ceritinib in patients with non-small-cell lung cancer harboring ROS1 rearrangement. J Clin Oncol. 2017;35:2612-2618. [PMID: 28520527]

Drilon A, Siena S, Dziadziuszko R, et al. Entrectinib in ROS1 fusion-positive non-small-cell lung cancer: integrated analysis of three phase 1-2 trials. Lancet Oncol. 2020;21(2):261-270. [PMID: 31838015]

Shaw AT, Solomon BJ, Chiari R, et al. Lorlatinib in advanced ROS1-positive non-small-cell lung cancer: a multicentre, open-label, single-arm, phase 1-2 trial. Lancet Oncol. 2019;20(12):1691-1701. [PMID: 31669155]

Chen H, Zhang Q, Zhang Y, et al. Afatinib reverses ceritinib resistance (CR) in ALK/ROS1-positive non-small-cell lung cancer cell (NSCLC) via suppression of NRG1 pathway. Onco Targets Ther. 2018;11:8201-8209. [PMID: 30568455]

Ku BM, Bae YH, Lee KY, et al. Entrectinib resistance mechanisms in ROS1-rearranged non-small cell lung cancer. Invest New Drugs. 2020;38(2):360-368. [PMID: 31124056]

Iyer G, Hanrahan AJ, Milowsky MI, et al. Genome sequencing identifies a basis for everolimus sensitivity . Science. 2012; 338(6104): 221-221. [PMID: 22923433]

Chung HC, et al First-line pembrolizumab/placebo plus trastuzumab and chemotherapy in HER2-positive advanced gastric cancer: KEYNOTE-811. Future Oncol 2021 Feb;17(5):491-501. [PMID: 33167735]

Hope S Rugo, et al. Efficacy of Margetuximab vs Trastuzumab in Patients With Pretreated ERBB2-Positive Advanced Breast Cancer: A Phase 3 Randomized Clinical Trial[J]. JAMA Oncol. 2021 Apr 1;7(4):573-584. [PMID: 33480963]

Reichardt P, Demetri G D, Gelderblom H , et al. Correlation of KIT and PDGFRA mutational status with clinical benefit in patients with gastrointestinal stromal tumor treated with sunitinib in a worldwide treatment-use trial[J]. Bmc Cancer, 2016, 16(1):1-10. [PMID: 26772734]

Blay J Y, Serrano C, Heinrich M C , et al. Ripretinib in patients with advanced gastrointestinal stromal tumours (INVICTUS): a double-blind, randomised, placebo-controlled, phase 3 trial[J]. The Lancet Oncology, 2020, 21(7). [PMID: 32511981]

Mensenkamp A R, Vogelaar I P, van Zelst–Stams, Wendy A.G, et al. Somatic Mutations in MLH1 and MSH2 Are a Frequent Cause of Mismatch-Repair Deficiency in Lynch Syndrome-Like Tumors[J]. Gastroenterology, 2014, 146(3):643-646.e8. [PMID: 24333619]

Haraldsdottir S, Hampel H, Tomsic J, et al. Colon and Endometrial Cancers With Mismatch Repair Deficiency Can Arise From Somatic, Rather Than Germline, Mutations[J]. Gastroenterology, 2014, 147(6):1308-1316.e1. [PMID: 25194673]

Overman M J, Mcdermott R, Leach J L, et al. Nivolumab in patients with metastatic DNA mismatch repair-deficient or microsatellite instability-high colorectal cancer (CheckMate 142): an open-label, multicentre, phase 2 study[J]. Lancet Oncology, 2017, 18(9):1182-1191. [PMID: 28734759]

Zhou C, Ramalingam SS, Kim TM, et al. Treatment Outcomes and Safety of Mobocertinib in Platinum-Pretreated Patients With EGFR Exon 20 Insertion-Positive Metastatic Non-Small Cell Lung Cancer: A Phase 1/2 Open-label Nonrandomized Clinical Trial. JAMA Oncol. 2021;7(12):e214761. [PMID: 34647988]

Zhao H, Yao W, Min X, et al. Apatinib Plus Gefitinib as First-Line Treatment in Advanced EGFR-Mutant NSCLC: The Phase III ACTIVE Study (CTONG1706). J Thorac Oncol. 2021;16(9):1533-1546. [PMID: 34033974]

Lu J, Zhong H, Chu T, et al. Role of anlotinib-induced CCL2 decrease in anti-angiogenesis and response prediction for nonsmall cell lung cancer therapy. Eur Respir J. 2019;53(3):1801562. Published 2019 Mar 7. [PMID: 30578392]
